# Applying a novel approach to scoping review incorporating artificial intelligence: mapping the natural history of gonorrhoea

**DOI:** 10.1186/s12874-021-01367-x

**Published:** 2021-09-06

**Authors:** Jane Whelan, Mohammad Ghoniem, Nicolas Médoc, Mike Apicella, Ekkehard Beck

**Affiliations:** 1 GSK, Amsterdam, The Netherlands; 2 Luxembourg Institute of Science and Technology, Esch-sur-Alzette, Luxembourg; 3 University of Iowa, Iowa City, USA; 4 GSK, Wavre, Belgium

**Keywords:** Scoping review, Artificial intelligence, Visual text mining, Natural language processing, Co-clustering, Clinical outcomes, Health problems, Gonorrhoea, *N. gonorrhoeae*, Natural history

## Abstract

**Background:**

Systematic and scoping literature searches are increasingly resource intensive. We present the results of a scoping review which combines the use of a novel artificial-intelligence-(AI)-assisted Medline search tool with two other ‘traditional’ literature search methods. We illustrate this novel approach with a case study to identify and map the range of conditions (clinical presentations, complications, coinfections and health problems) associated with gonorrhoea infection.

**Methods:**

To fully characterize the range of health outcomes associated with gonorrhoea, we combined a high yield preliminary search with a traditional systematic search, then supplemented with the output of a novel AI-assisted Medline search tool based on natural language processing methods to identify eligible literature.

**Results:**

We identified 189 health conditions associated with gonorrhoea infection of which: 53 were identified through the initial ‘high yield’ search; 99 through the systematic search; and 124 through the AI-assisted search. These were extracted from 107 unique references and 21 International Statistical Classification of Diseases and Related Health Problems Ninth and Tenth Revision (ICD 9/10) or Read codes. Health conditions were mapped to the urogenital tract (*n* = 86), anorectal tract (*n* = 6) oropharyngeal tract (*n* = 5) and the eye (*n* = 14); and other conditions such as systemic (*n* = 61) and neonatal conditions (*n* = 7), psychosocial associations (*n* = 3), and co-infections (n = 7). The 107 unique references attained a Scottish Intercollegiate Guidelines Network (SIGN) score of ≥ 2++ (*n* = 2), 2+ (14 [13%]), 2- (30 [28%]) and 3 (45 [42%]), respectively. The remaining papers (*n* = 16) were reviews.

**Conclusions:**

Through AI screening of Medline, we captured – titles, abstracts, case reports and case series related to rare but serious health conditions related to gonorrhoea infection. These outcomes might otherwise have been missed during a systematic search. The AI-assisted search provided a useful addition to traditional/manual literature searches especially when rapid results are required in an exploratory setting.

**Supplementary Information:**

The online version contains supplementary material available at 10.1186/s12874-021-01367-x.

## Background

**Fig. 1:**
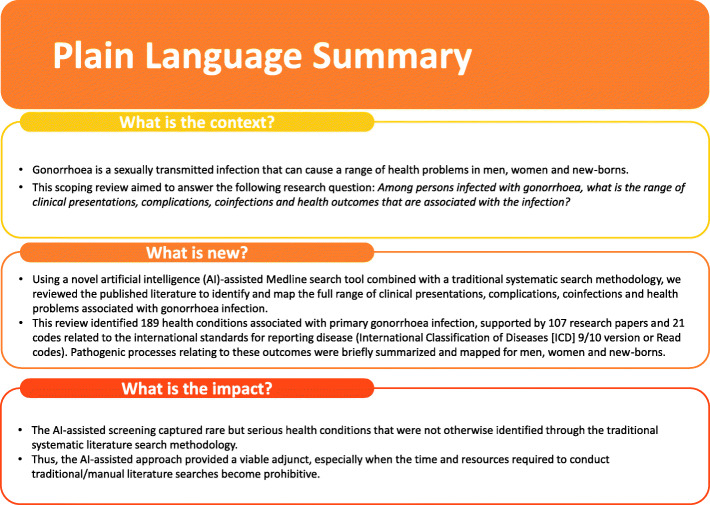
Plain Language Summary

In recent years, scoping review methodologies have emerged as an alternative to systematic reviews if the objective is more exploratory in nature [1, 2]. Scoping reviews allow us to map key concepts or definitions in specific research areas, identify and analyse research knowledge gaps, or examine the nature of available evidence in a given field. While systematic review methodology is the gold standard to synthesize empirical evidence and address a discrete research question in a reproducible manner, scoping reviews address different objectives and review more complex or heterogeneous literature [2]. Both methods share common standards, such as: the use of systematic methods and the comprehensive nature of the search. However, as the volume of published literature increases exponentially [3], these methods are also increasingly resource intensive and expensive to conduct. Simultaneously, novel artificial intelligence (AI) assisted technologies are emerging with the potential to aid in literature screening in an efficient manner. In this manuscript, we report the use of such an AI-assisted Medline search tool which was combined with a traditional systematic search methodology in a scoping review. This paper is intended to provide the reader with a practical illustration of the combined method described above when applied to a clinical research question: in this case, related to sexually transmitted disease. Our objective was to identify and map the clinical presentations, complications, coinfections and health problems that have been associated with gonorrhoea infection as identified in published literature, while trialling an AI-assisted literature search tool combined with traditional search methods in a scoping review. Please refer to Fig. 1 for a plain language summary of this study.

### Case study: Gonorrhoea infection

Gonorrhoea, caused by the bacterium *N. gonorrhoeae (Ng),* is a sexually transmitted disease that has established resistance to all known antibiotics developed since the 1930s, and the World Health Organization (WHO) recently warned that the emergence of pan-drug resistant *Ng* is on the horizon [4]. In the US alone, almost half of the estimated 1.14 million cases occurring each year are resistant to antibiotics. If gonorrhoea becomes untreatable, its associated complications and health problems could result in an additional 1.2 million *Ng* infections and 579 gonorrhoea-attributable human immunodeficiency virus (HIV) infections within 10 years at a cost of $378.2 million [5].

The course of gonorrhoea infection within an individual over time, known as the natural history, is highly complex and involves multiple anatomic sites of primary mucosal infection in men, women and neonates [6]. Anatomic sites include the urogenital, anorectal and pharyngeal tract, as well as the eye, particularly in neonates. Infection with *Ng c*an directly result in a wide spectrum of clinical presentations and complications, and is indirectly associated with a range of other health problems in the short, medium and long-term [7]. Some complications and/or health problems are well described in the literature, such as pelvic inflammatory disease (PID) and infertility in women and epididymitis in men [6, 8]. However, the full range of conditions associated with gonorrhoea has not been systematically characterized in the literature in an accessible, evidence-based format.

## Methods

### Overview of scoping review design

**Fig. 2:**
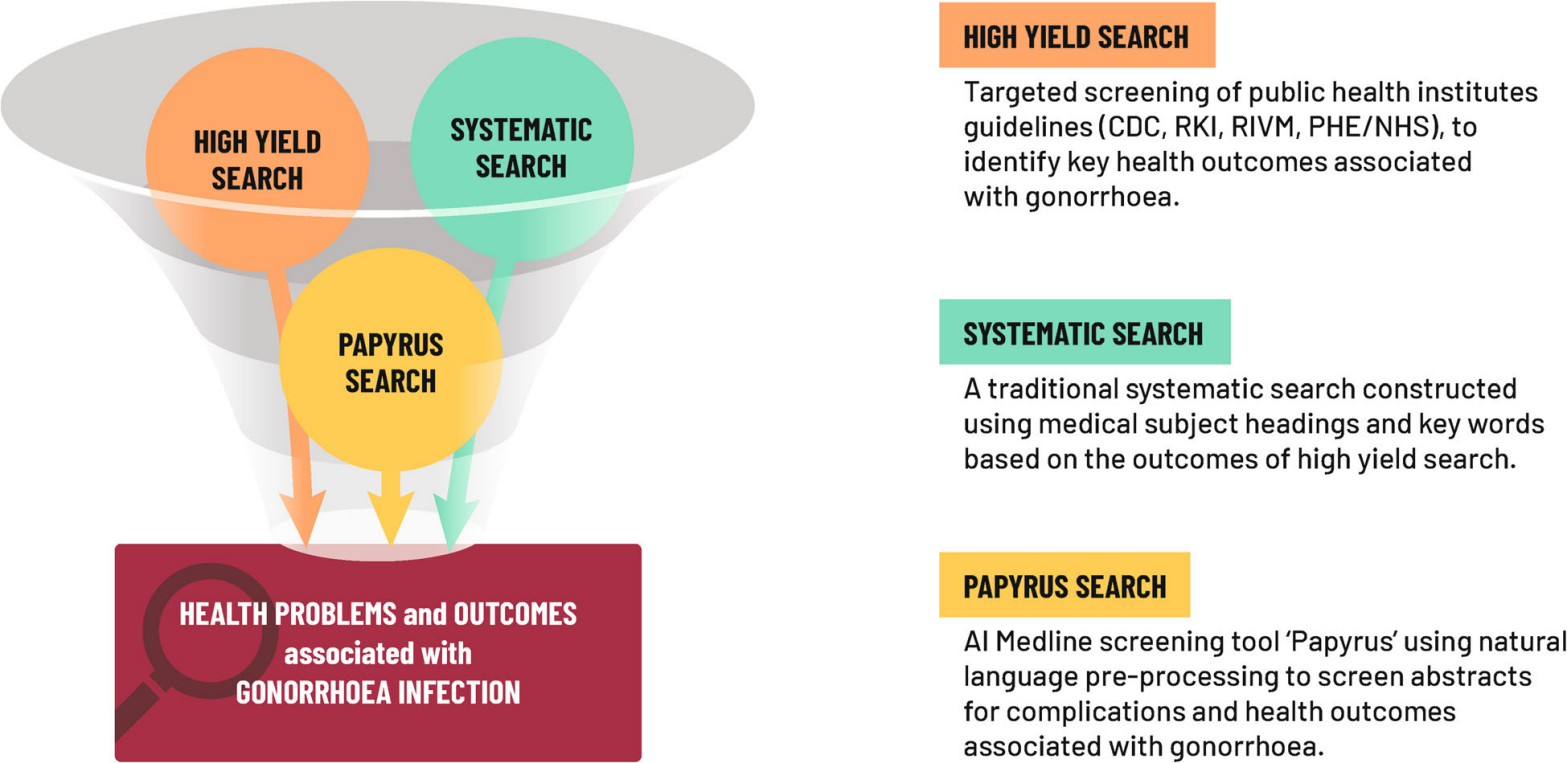
The traditional systematic search methodology was combined with an innovative AI-assisted Medline screening. AI, artificial intelligence; CDC, Center for Disease Control and Prevention; PHE/NHS, Public Health England/National Health Service, England; RIVM, National Institute for Public Health and Environment, Netherlands; RKI, Robert Koch Institute, Germany

Due to the exploratory nature of the research question, we anticipated that the traditional ‘population, intervention, comparator and outcome’ (PICO) [9] -based formulation of the research question using systematic review methods alone would be either too restrictive (gonorrhoea-related conditions would have to be specified a priori*,* limiting the outcome), or alternatively, too imprecise, resulting in a high number of hits of low specificity. To address this, we used an established scoping review methodology [2] combining three complementary approaches to search the published literature. These included a ‘high yield’ preliminary search combined with a traditional systematic search, then supplemented with the output of a novel AI-assisted Medline search (Fig. 2), which we report here for the first time. The ‘high yield’ search screened public health institute websites such as those of the Centre for Disease Control and Prevention (CDC), Public Health England/National Health Service, England (PHE/NHS), British Association for Sexual Health and Human Immunodeficiency Virus (BAHH), National Institute for Public Health and Environment, Netherlands (RIVM) and Robert Koch Institute, Germany (RKI) [10–14]. The systematic literature search combined Medical Subject Headings (MeSH terms) with keywords to screen for relevant publications. Lastly, the AI-assisted Medline search used a natural language pre-processing tool called Papyrus [15], to screen abstracts for ‘topic words’ related to gonorrhoea (Additional file 1).

### Formulation of research question

The research question was based on the ‘patient, concept and context (PCC) structure [16], for scoping reviews: ‘*Among persons infected with gonorrhoea, what is the range of clinical presentations, complications, coinfections and health outcomes that are associated with the infection?* Identified conditions were then contextualized according to known pathogenic processes, to be associated with primary urogenital, anorectal, oropharyngeal or conjunctival infection. Identified papers were eligible for inclusion if they described a potential association between primary gonorrhoea infection at any anatomic site with any clinical or psychosocial health outcome in women, men or children. In turn, ‘association’ was assumed to imply that gonorrhoea infection could be a plausible component along the causal pathogenic pathway to the health outcome, either directly or indirectly [17, 18]. All study designs (including case reports and case series) were eligible for inclusion.

### Search strategy

Three distinct search strategies were employed: a ‘high yield search’, traditional systematic search and a search using an AI-assisted Medline search tool (Fig. 2).

Search 1: To compile an initial list of key health problems associated with *Ng* infection, we first conducted a ‘high yield’ or ‘snowball’ search [19], accessing websites of major public health institutes in the United States (US), United Kingdom (UK), Germany and the Netherlands [10–14] to review current disease summaries and guidance on gonorrhoea (Supplementary text 2.1, Additional file 2). The search was conducted (by JW and EB), over the month of September, 2019. We pursued targeted Medline searches of key health problems based on the quoted references, knowledge of seminal authors and studies in the field, and the reference list of each paper (Supplementary text 2.2, Additional file 2). The resulting list of health problems was then compared against the existing compendia of clinical diagnoses related to gonorrhoea from the International Statistical Classification of Diseases and Related Health Problems Ninth and Tenth Revision (ICD9/10 [20, 21] and Read diagnostic codes [22]) (Supplementary Table 1–2, Additional file 2). The list of health problems was supplemented with ICD codes (used to systematically classify diseases, disorders, injuries and other health conditions) where necessary.

Search 2: We then conducted a traditional systematic Medline search applying the PICO methodology [9], posing the broad question “*In people exposed to Ng, what is the natural history of gonorrhoea infection?”* The search string was developed iteratively (by JW and EB, and applied on 04 November, 2019) (Additional file 3), combining keywords and MeSH terms identified from seminal references which in-turn resulted from the snowball search. Full-text articles were retrieved if the title and abstract specifically related aspects of the natural history or pathogenesis of *Ng* to clinical sequelae or health problems in humans. Only English language abstracts were included. No other limitations were applied. Reference lists were reviewed and full-text articles were accessed where relevant. The outcome of this search was used to provide a brief narrative summary of the key pathogenic processes associated with complications and health problems identified as well as to identify further health problems associated with *Ng.*

**Fig. 3:**
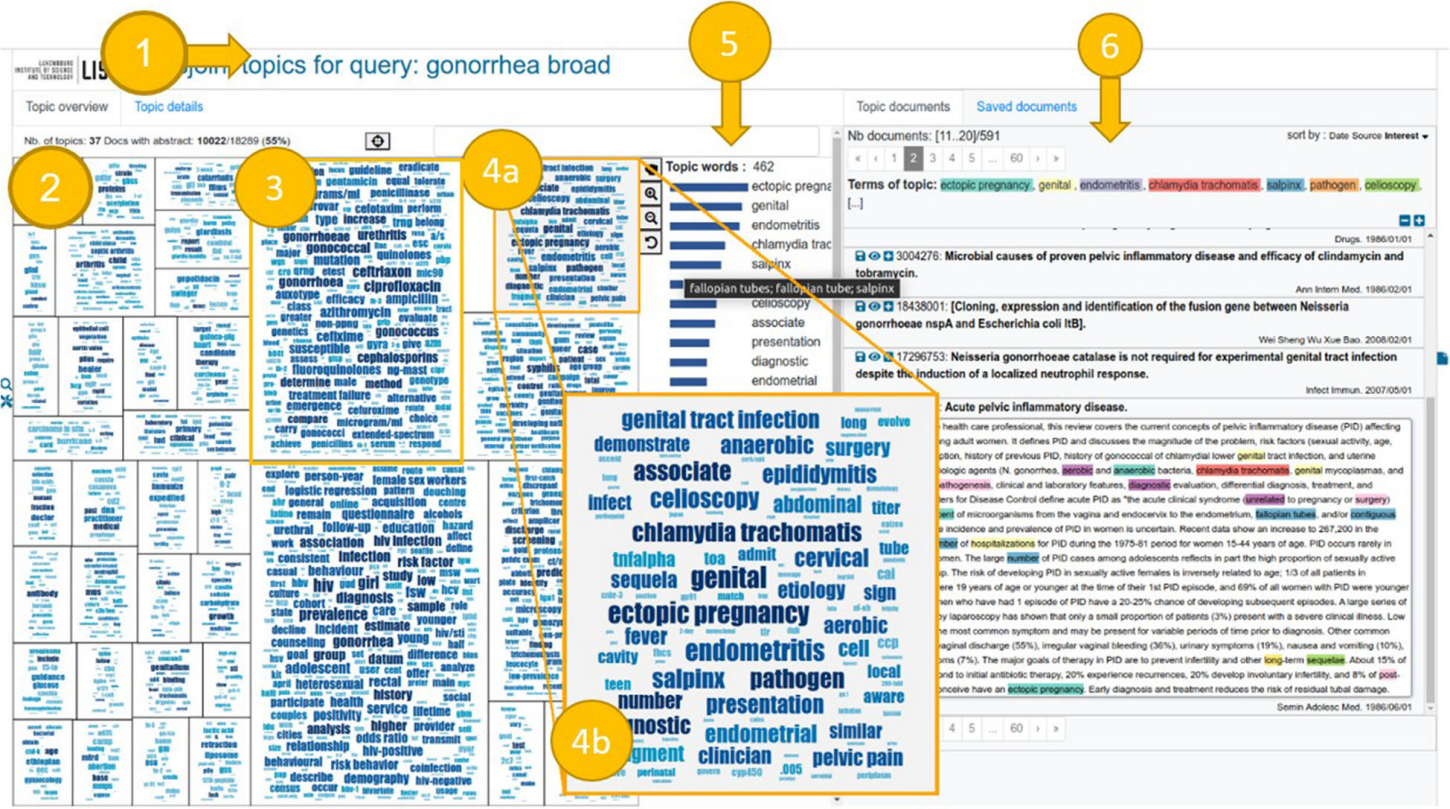
Overview of the corpus in the Papyrus software^±^. ^±^Overview of the corpus in the Papyrus software. (1) The broad search query ‘gonorrhea’ is entered in the search box. (2) Topics related to the search query ‘gonorrhea’ are extracted automatically from the papyrus corpus and presented as a mosaic of rectangles on screen. In the present case, the map contains 37 distinct topics (rectangles) of inter-related words that were extracted by the tool. (3) A highlighted example is a topic related to antibiotics including topic words such as ‘cephalosporins’, ‘ciprofloxacin’, ‘ceftriaxon’. By glancing over the combination of words shown in each rectangle, the user is able to infer whether the topic captures subject matter relevant to the search query (i.e., a subjective evaluation on behalf of the user). (4) When the user clicks on a topic of interest (e.g., 4a the rectangle containing ‘ectopic pregnancy’ [4a]) a ranked list of inter-related ‘topic words’ is displayed based on their relative importance to the topic at hand (e.g., ‘endometritis’, ‘epididymitis’ and ‘salpinx’ appear in the top 5 commonly occurring topic-words [inset, 4b]). (5) The chosen topic is then shown to comprise a total of 462 representative topic-specific words, presented according to their relative frequency of occurrence. (6) Abstracts from each of the topic words (in this case 591 abstracts) can then be displayed, with direct link to PubMed provided

Search 3: We supplemented the searches with Papyrus [15], a novel AI-assisted Medline search tool, which is described in detail in Supplementary text 4.1–4.3, and Additional file 4). A broad search query (‘gonorrhoea [All Fields]’) was run on July 5, 2019, identifying relevant literature with an English title and abstract. The AI tool used automatic natural language processing (NLP) methods and pre-processing using the Stanford Core NLP library [23] (see details in Supplementary text 4.1, Additional file 4) to extract identified ‘topic-words’ from all abstracts – typically nouns or expressions describing concepts related to gonorrhoea (e.g. ‘salpinx’ or ‘ectopic pregnancy’). A vector space model was constructed and a ‘CoClus’ co-clustering algorithm [24] (see details in Supplementary text 4.2, Additional file 4) was applied to partition the vocabulary and the document set into topics, so that each topic comprises semantically related ‘topic-words’ and their enclosing documents (e.g. an analogy in the press would be to discover automatically without prior knowledge a topic where some of the most important words are ‘covid19’, ‘lockdown’, ‘mask’, ‘PCR’, ‘vaccine’, ‘test’, ‘layoff’, ‘stimulus’, ‘bill’). Within each topic, associated ‘topic words’ are ranked by a score based on the frequency with which these words occur in abstracts, reflecting their importance with respect to the given topic (Supplementary text 4.1, Additional file 4). Supplementary Fig. 1, Additional file 2 shows an example of the raw textual output of the words listed under a topic, as extracted by the tool. Figure 3 shows the graphical user interface of Papyrus, which is composed of a topic map in the form of a mosaic of word clouds. It illustrates how each rectangle is a topic grouping a subset of abstracts (e.g., outcomes related to urogenital *Ng* infections) and their most representative topic-words (‘ectopic pregnancy’, ‘endometritis’, ‘epididymitis’ and ‘salpinx’). Details of the NLP methods are provided in Supplementary text 4.1, Additional file 4. As a first step, all ‘topic-words’ corresponding to each topic displayed in the topic map were extracted and screened manually and independently by two reviewers (JW and EB) for relevance to clinical and psychosocial gonorrhoea-related health outcomes. The papers corresponding to the agreed topic-words were then manually screened and full-text articles were only accessed if the inclusion criteria were met.

### Data synthesis

To map the list of health problems and outcomes associated with *Ng* infections, the three approaches were cross referenced, duplicate conditions and references were removed and reported according to the Preferred Reporting Items for Systematic reviews and Meta-Analyses extension for Scoping Reviews (PRISMA-ScR) [25]. The retrieved reports were categorized by study design (e.g., cohort study/case control/literature review [Supplementary text 5.1, Additional file 5]) and primary research papers were assigned a quality score according to the Scottish Intercollegiate Guidelines Network (SIGN) criteria [26]. As some health outcomes are serious but rare (e.g., disseminated gonorrhoea infection [DGI]), categories of evidence included case reports, case series (SIGN score of 3) and higher levels of evidence. Health outcomes identified through secondary reporting in review papers only (and not in primary research) were also included as we considered that primary research from the pre-antibiotic era may not have been indexed on PubMed. Where associated conditions were derived from the clinical compendia of ICD 9/10 or Read codes (classification of clinical terms for describing the care and treatment of patients), these were categorized separately based on the causal pathogenic pathway. To summarize the results, health outcomes with the highest level of supporting evidence (SIGN score) were selected for inclusion in an illustrative figure. The full evidence table was reviewed by an independent expert (MA) for the plausibility of association with gonorrhoea, based on the known pathogenesis of the infection. All identified conditions, associated references, study design and SIGN scores are provided in Supplementary text 5.1, Additional file 5.

## Results

**Fig. 4:**
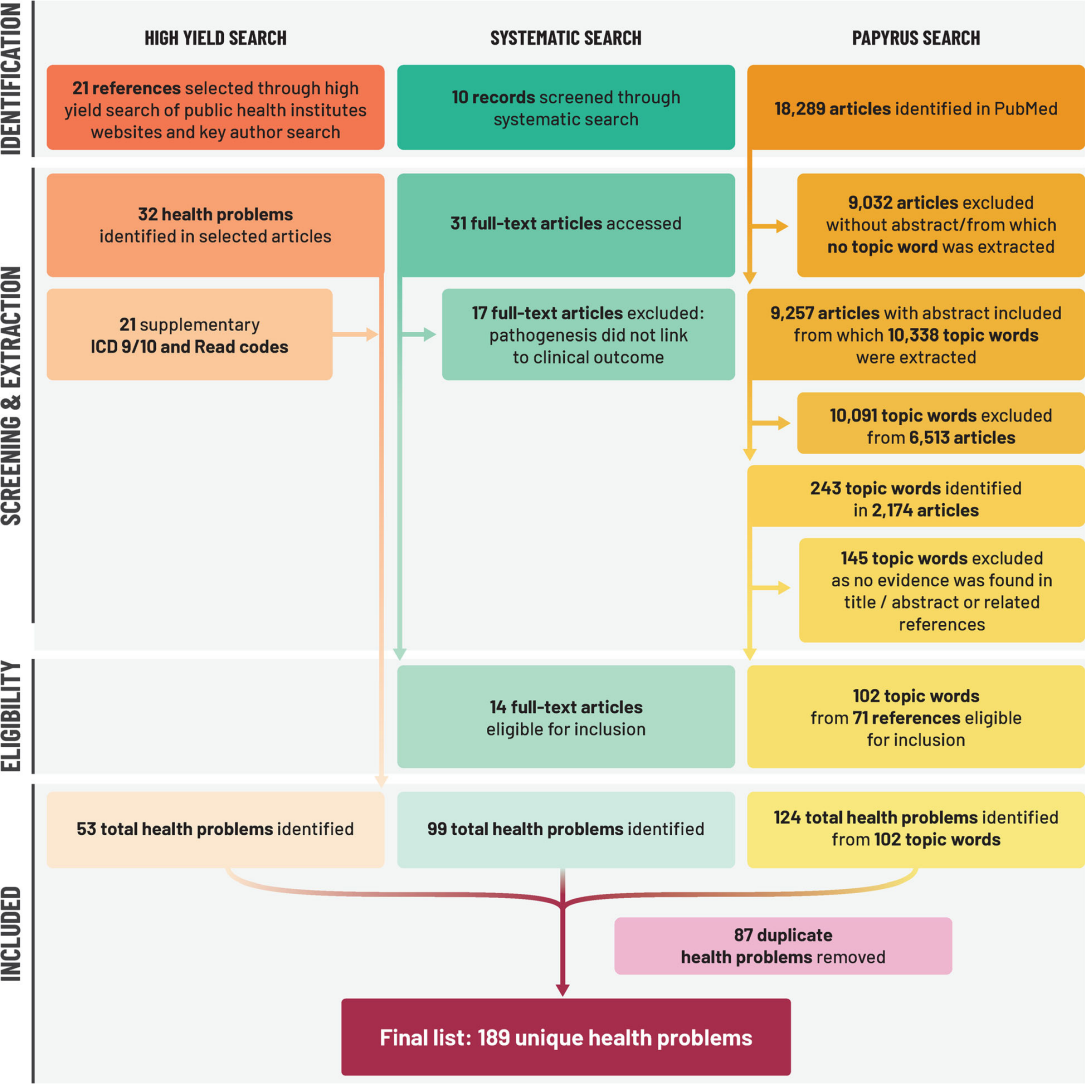
PRISMA-ScR flowchart. ICD 9/10, International Statistical Classification of Diseases and Related Health Problems (Ninth or Tenth Revision); PRISMA-ScR, Preferred Reporting Items for Systematic reviews and Meta-Analyses extension for Scoping Reviews

**Fig. 5:**
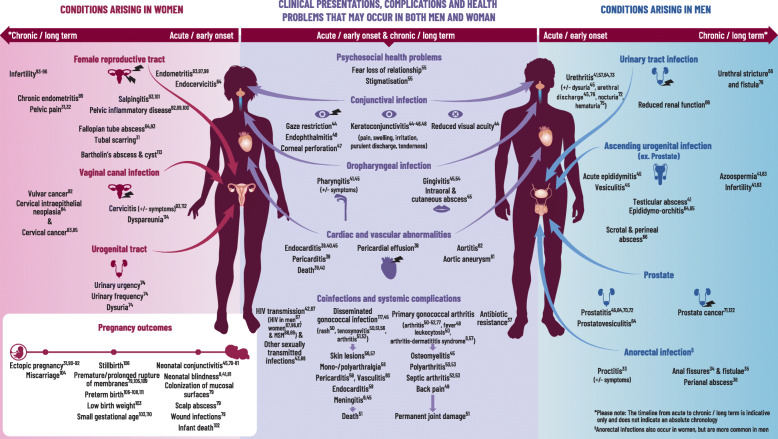
Gonorrhoea health map: Clinical presentations, complications and health problems that may occur in both men and women. *Neisseria gonorrhoeae* (*Ng*) is transmitted person-to-person through sexual networks by direct contact between mucosal surfaces of the urogenital, anorectal or oropharyngeal tracts, and sometimes via the eye. In men, it attaches to the sperm and is transmitted via the ejaculate to their partners (50–73% probability, independent of number of exposures) [6]. In women, enzymes in the cervicovaginal flora facilitate transfer to and uptake of *Ng* by the male urethra (20–35% probability with one exposure) [6]. One third of exposures will not result in infection but in the remainder, the incubation period is 1–6 days [27]. In both sexes, the first step in the pathogenesis is adherence to the epithelium of the human mucosal surface. In the urogenital tract, *Ng* enters male and female epithelial cells through different receptors, leading to different clinical presentations (i.e., cervicitis in women, urethritis in men) [6]. For most women, Ng infection of the lower genital tract is asymptomatic but sub-clinical cervicitis can cause reproductive sequelae over time [28]. In both men and women, symptomatic infection results from a local influx of neutrophils and production of inflammatory mediators. The *Ng* bacterium evades the innate immune response and manipulates the adaptive immune response to promote continued inflammation [29]. This facilitates sub-epithelial penetration associated with increased susceptibility to human immunodeficiency virus type 1 [6]. Without treatment, *Ng* can ascend the urogenital tract. Intense neutrophilic activity in the upper tract directly damages epithelial cells [29] and leads to the death of cells lining the upper tract. Subsequently this may cause scarring and occlusion (e.g., causing tubal factor infertility, ectopic pregnancy in women and urethral stricture in men) [30]. In women, inflammation and intra-abdominal adhesions have also been associated with chronic pelvic pain [31]. If *Ng* enters the bloodstream and disseminates, interacting with other host cell types (e.g., blood vessel endothelial dendritic cells, macrophages), it may cause skin and/or joint/tendon infection, and more rarely endocarditis or meningitis and other systemic sequelae [32]. Pregnant women can transmit *Ng* to their newborns during delivery, which may result in neonatal conjunctivitis and/or rarely, disseminated infection. A comprehensive summary can be seen in the accompanying fig [31–114].

References were identified through each of the three searches and are fully detailed in Fig. 4. After screening for eligibility according to the inclusion criteria we identified, 53 health conditions through the initial ‘high yield’ search, 99 through the systematic search and 124 (from 102 topic words) through the AI-assisted search. Details of the search output in terms of ‘topic words’ and related abstracts for the AI-tool are also provided in Fig. 4 and supplementary text 5.2, Additional file 5. After the removal of duplicate conditions (*n* = 87), the search resulted in a total of 189 health conditions associated with primary gonorrhoea infection, supported by 107 unique references and 21 ICD 9/10 or read codes (Fig. 4). Pathogenic processes relating to these outcomes were briefly summarized based on 14 out of 101 full-text articles retrieved during the systematic review (Additional file 3 and Fig. 5) to provide context to the output. We applied no search restrictions in terms of sex or gender, but all manuscripts identified reported on either men (including MSM) and/ or women only. For a graphical representation of the range of health problems identified in the upper urogenital and reproductive tracts, conditions were assigned to either men or women based on the anatomy of the upper urogenital and reproductive tracts, respectively (Fig. 5). Each condition was attributed to the likely site of primary infection, and whether it was a primary clinical presentation/symptom, or a related complication, coinfection or long-term health outcome. Among the identified conditions, 86 were related to primary urogenital infection, 6 were related to the anorectal tract, 5 were related to oropharyngeal infection, 14 were infections in the eye, 3 were psychosocial and 7 were in neonates. In some cases (*n* = 61) the condition was systemic in nature and was not directly attributable to a single primary site of infection. Lastly, 7 conditions were classified as co-infections (e.g., HIV). Furthermore, studies related to these 189 conditions utilized various study designs, which included 132 primary research studies, 56 clinical/microbiological reviews, and the remainder were ICD 9/10 or Read codes. We identified a total of 107 supporting research papers, among which only 2 papers were SIGN ≥2++; 14 (13%) of SIGN score 2+, 30 (28%) of SIGN score 2- and 45 (42%) of SIGN score of 3 were found. The remaining 16 papers were reviews. To illustrate our findings, a subset of conditions and the highest quality of related references were selected for inclusion in Fig. 5. The full list of conditions and sources is provided in supplementary text 5.1, Additional file 5. Of the references identified, 50% were published before 2004 and 25% before 1991

## Discussion

The case study on which this scoping review was based, allowed us to trial an innovative methodology while also answering an important clinical research question. More than 580,000 gonorrhoea infections were notified in the US in 2018, the highest number reported since 1991 representing an increase of 83% since the historic low of 2009 [115]. As the range of effective antimicrobial therapies is depleted over time, the prospect of untreatable gonorrhoea is increasing [116]. Health outcomes that are rare or unseen in the contemporary industrialized world may again become commonplace [117], with serious implications for public health and healthcare costs [118]. To fully characterize the range of health outcomes associated with gonorrhoea, we combined traditional and novel literature searching methodologies. As we had anticipated, less than half of the conditions identified were captured through the ‘high yield’ and systematic searches combined, and the AI tool added substantial value in this regard. Through AI screening of Medline titles and abstracts, case reports and case series (in particular) that related to rare but serious health conditions were captured. These outcomes might otherwise have been missed during a systematic search. Such non-analytic studies score low on objective quality scores but relate to conditions that may be important contributors to the total burden of gonorrhoea infection if gonorrhoea becomes untreatable in the future. For some outcomes, where primary research was not identified because it may have been conducted in the pre-PubMed era, we refer to review articles as secondary references. In the 1930s for example, 140 cases of gonococcal arthritis associated with 3 deaths were described over a 6-year period at Boston City Hospital [119, 120]. Such health outcomes, that commonly occurred in the pre-antibiotic and/or pre-PubMed era but are rare today in the industrialized world, may not otherwise have been identified.

There were limitations to our study. Similar to a manual systematic search, the AI tool returned topic words with a related abstract that required independent manual review and it is possible that some important health conditions were missed. We reviewed only English language abstracts, hence health problems reported in the full-text articles may not have been mentioned in the abstract. It is also possible that the AI approach was overly sensitive, identifying health problems that are excessively rare (e.g., Adult respiratory distress syndrome) [121] and not likely to re-emerge, or for which a direct causal association may not be clearly established (e.g., prostate cancer) [122]. Finally, we made no selection based on sex or gender, but all identified complications and health outcomes were attributable only to men and/or women in the literature referenced. The scope of returned abstracts did not report on the health effects of non-binary, intersex or transgender people. This may suggest that, overall, the literature on the health effects of gonorrhoea is limited, also with regards to sexes. This remains an important research gap yet to be addressed.

To our knowledge, this scoping review remains the most comprehensive search, selection and synthesis of health conditions that has been related to gonorrhoea. Notably, the volume of high-quality research on the health outcomes of gonorrhoea was limited, and few controlled, observational studies with a low risk of confounding or bias that estimated a causal association were identified.

In conclusion, this scoping review using combined search methods proved to be a useful starting point from which to quickly but comprehensively identify relevant papers, inform future systematic literature searches, identify gaps in the existing literature and formulate new research questions. In our experience, AI-assisted Medline screening facilitated the exploratory nature of our research question and identified valuable supplementary material. Considering the exponential growth in the scientific literature [3], traditional/manual literature search methods will be limited in their future application as the time and resources required become prohibitive. The presented approach provides a viable adjunct, especially in situations where timely results of literature searches are of essence such as in the current situation with coronavirus disease (COVID-19) [123]. At the time of writing, the software developers are assessing the validity of the AI-assisted literature screening approach described in this work, so that it can be applied to any systematic literature review work. Specifically, Papyrus is being used to write a survey paper in computer science on the topic of “data visualization on large high-resolution displays”. This work in progress is based on six relevant scientific databases (Springer, Wiley Online Library, ScienceDirect, ACM digital library, IEEE Xplore, EBSCO Host). The developers have also put in place an instance of the Papyrus software to help analyse the literature concerning the COVID19 disease, which can be accessed online at https://colibri.list.lu/. Provision of full support is planned for all the steps of the PRISMA workflow within the tool, e.g. integrated support for paper annotation and scoring.

Pending further testing and validation, AI-assisted literature searching has the potential to become an important tool in the existing lexicon.

## Supplementary Information

**Additional file 1: Supplementary Text 1.** Papyrus tutorial.

**Additional file 2: Supplemental Fig. 1.** Raw textual output listing a sample of representative words of one of the topics identified by the AI methods of the Papyrus tool from the corpus resulting from the search query ‘gonorrhea’. In this example, the most important topic-word (most frequently occurring) is ‘ectopic pregnancy’ followed by other words like ‘chlamydia trachomatis’ and ‘salpinx’. **Supplementary Text 2.1.** Public Health Websites accessed for the initial ‘high yield’ search. **Supplementary Text 2.2.** Seminal literature based on a review of key authors in the field. **Supplementary Table 1.** ICD9/ICD10 and Read (CPRD) codes. **Supplementary Table 2.** Read codes (level 3).

**Additional file 3: Supplementary Table 3.** Systematic search Strategy and number of records retrieved. **Supplementary Table 4.** Inclusions from systematic search.

**Additional file 4: Supplementary Text 4.1.** Description of the Papyrus system. **Supplementary text 4.2.** Interactive visualization. **Supplementary Text 4.3.** Reproducibility and General Applicability of this Approach.

**Additional file 5: Supplementary Text 5.1.** Full list of health outcomes identified through the combination of the AI and other searches, with references and SIGN scores. **Supplementary Text 5.2.** List of extracted ‘topic words’ related references and health outcomes identified through AI-assisted literature search.

## Data Availability

All data generated or analysed during this study are included in this published article and its supplementary information files.

## Abbreviations

AI: Artificial intelligence
COVID-19: Coronavirus disease
DGI: Disseminated gonorrhoea infection
HIV: Human immunodeficiency virus
ICD 9/10: International Statistical Classification of Diseases and Related Health Problems Ninth and Tenth Revision
MeSH: Medical Subject Headings
NLP: Natural language processing
*Ng*: *N. gonorrhoeae*
PID: Pelvic inflammatory disease
PICO: Population, intervention, comparator and outcome
PCC: Patient, concept and context
PRISMA ScR: Preferred Reporting Items for Systematic reviews and Meta-Analyses extension for Scoping Reviews (PRISMA-ScR)
SIGN: Scottish Intercollegiate Guidelines Network
TFI: Tubal factor infertility
US: United States
UK: United Kingdom
WHO: World Health Organization
