# Correction to: Applying a novel approach to scoping review incorporating artificial intelligence: mapping the natural history of gonorrhoea

**DOI:** 10.1186/s12874-021-01470-z

**Published:** 2021-12-12

**Authors:** Jane Whelan, Mohammad Ghoniem, Nicolas Médoc, Mike Apicella, Ekkehard Beck

**Affiliations:** 1 GSK, Amsterdam, The Netherlands; 2 Luxembourg Institute of Science and Technology, Esch-sur-Alzette, Luxembourg; 3 University of Iowa, Iowa City, USA; 4 GSK, Wavre, Belgium

**Correction to: BMC Med Res Methodol 21, 183 (2021)**

**https://doi.org/10.1186/s12874-021-01367-x**

Following publication of the original article [1], the authors noticed that the reference citations in Fig. 5 image were not aligned with the new references numbering. Presented here is the corrected Fig. 5 image. The original article has been updated.

**Fig. 5:**
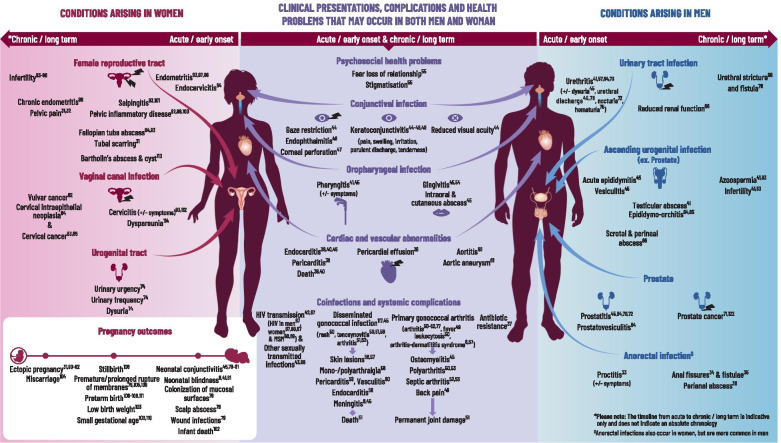
Gonorrhoea health map: Clinical presentations, complications and health problems that may occur in both men and women. *Neisseria gonorrhoeae* (*Ng*) is transmitted person-to-person through sexual networks by direct contact between mucosal surfaces of the urogenital, anorectal or oropharyngeal tracts, and sometimes via the eye. In men, it attaches to the sperm and is transmitted via the ejaculate to their partners (50–73% probability, independent of number of exposures) [6]. In women, enzymes in the cervicovaginal flora facilitate transfer to and uptake of *Ng* by the male urethra (20–35% probability with one exposure) [6]. One third of exposures will not result in infection but in the remainder, the incubation period is 1–6 days [27]. In both sexes, the first step in the pathogenesis is adherence to the epithelium of the human mucosal surface. In the urogenital tract, *Ng* enters male and female epithelial cells through different receptors, leading to different clinical presentations (i.e., cervicitis in women, urethritis in men) [6]. For most women, Ng infection of the lower genital tract is asymptomatic but sub-clinical cervicitis can cause reproductive sequelae over time [28]. In both men and women, symptomatic infection results from a local influx of neutrophils and production of inflammatory mediators. The *Ng* bacterium evades the innate immune response and manipulates the adaptive immune response to promote continued inflammation [29]. This facilitates sub-epithelial penetration associated with increased susceptibility to human immunodeficiency virus type 1 [6]. Without treatment, *Ng* can ascend the urogenital tract. Intense neutrophilic activity in the upper tract directly damages epithelial cells [29] and leads to the death of cells lining the upper tract. Subsequently this may cause scarring and occlusion (e.g., causing tubal factor infertility, ectopic pregnancy in women and urethral stricture in men) [30]. In women, inflammation and intra-abdominal adhesions have also been associated with chronic pelvic pain [31]. If *Ng* enters the bloodstream and disseminates, interacting with other host cell types (e.g., blood vessel endothelial dendritic cells, macrophages), it may cause skin and/or joint/tendon infection, and more rarely endocarditis or meningitis and other systemic sequelae [32]. Pregnant women can transmit *Ng* to their newborns during delivery, which may result in neonatal conjunctivitis and/or rarely, disseminated infection. A comprehensive summary can be seen in the accompanying fig [31–114]
