# Supplementary material for: Applying a novel approach to scoping review incorporating artificial intelligence: mapping the natural history of gonorrhoea
Source: BMC Med Res Methodol. 2021 Sep 6;21:183. doi: 10.1186/s12874-021-01367-x (PMC8418964; doi:10.1186/s12874-021-01367-x)

# Additional File 1

1. To start using Papyrus, browse the link <https://papyrus.list.lu> and access the application using the “guest login”.


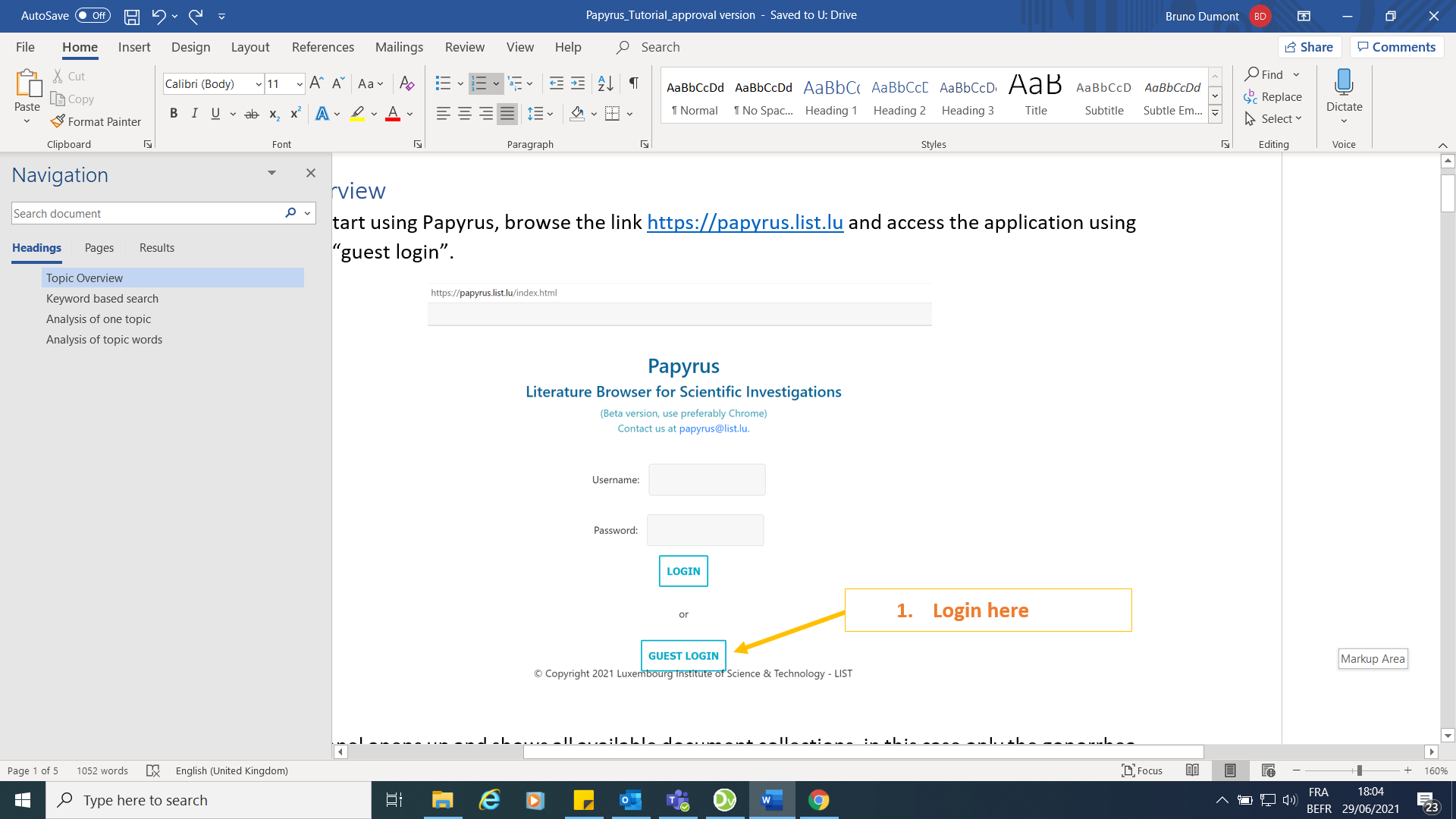


1. A panel opens up and shows all available document collections which have previously been processed by the system (using Natural Language Processing and extraction of keywords and topics). In this case only the gonorrhoea topic is presented
2. Beside the gonorrhoea collection, the number of abstracts related to the total number of titles identified is reported in blue (in the case of gonorrhoea, 10022 abstracts from a total of 18289 titles (i.e. 9.032 articles without an abstract were excluded, as reported in figure 3 in the paper).
3. Once the user has clicked ‘load’ on the desired collection, a visual summary of the corpus appears in the form of a rectangular cartogram/map of topics. This is displayed in the ‘topic overview’ tab. Of note, there is also a ‘topic details’ tab', but as this function was not utilised for this study, we do not describe this function in detail here.


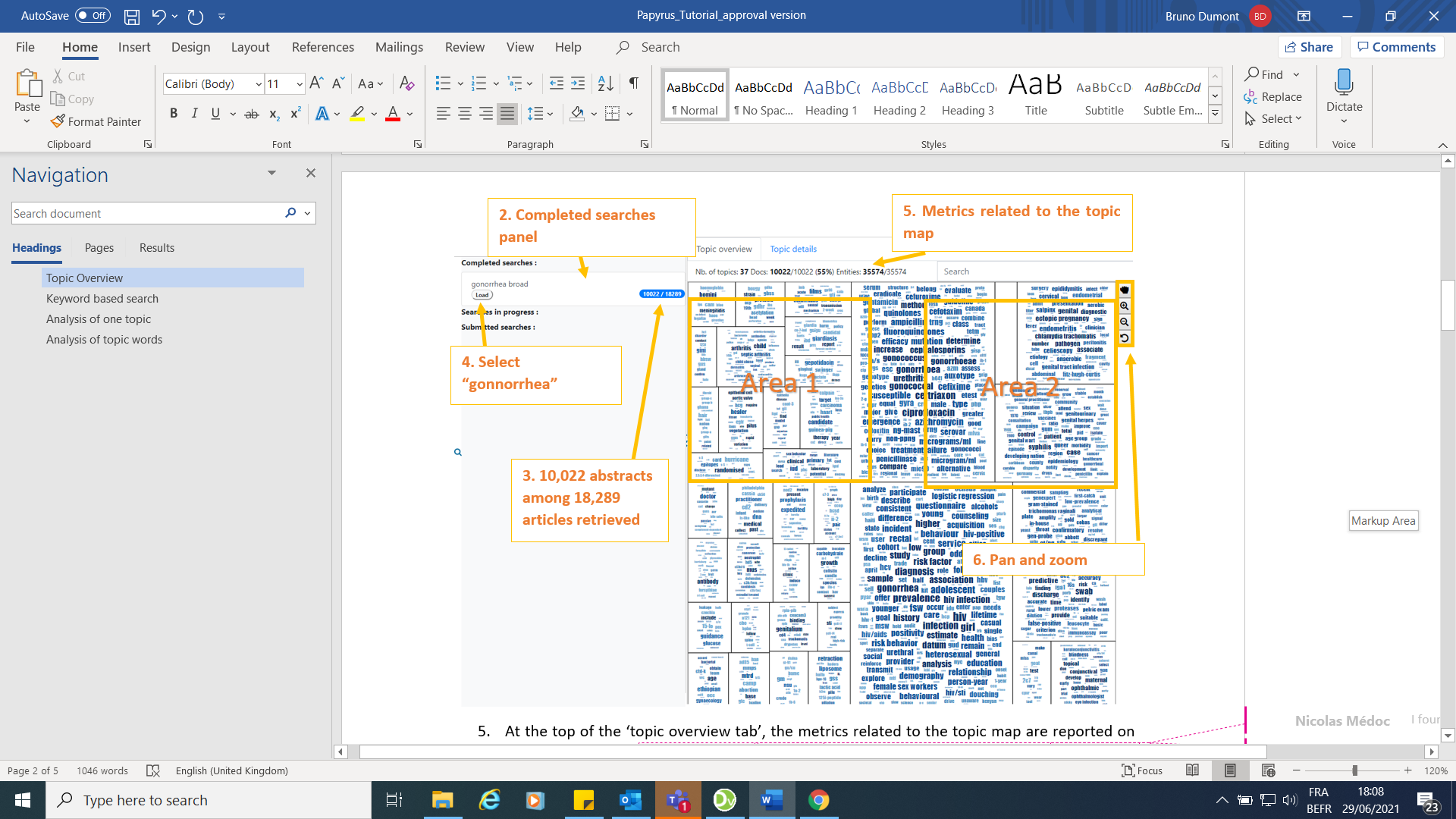


1. At the top of the ‘topic overview tab’, the metrics related to the topic map are reported on the top of the map. Within the entire collection, 10,022 documents are identified, from which 35,574 ‘entities’ or ‘key words’ are extracted, which are further explained below. In this case, the topic map is composed of 37 rectangles/tiles, each representing one topic i.e. a cluster of articles and their most related keywords extracted with a co-clustering or topic modelling algorithm. The ‘disjoint’ clustering approach assigns each topic word and related article to one and only one topic. The words belonging to each topic are visualized as a tag cloud where the font size and the colour encode the importance of words. By examining each tag cloud, the user can figure out the meaning carried by each topic/tile, and the relevance to their research question.

1. Like in popular geographic map software, the user can pan and zoom the topic map by using the magnifying lens on the right to better explore each topic individually (see Area 1 and 2).


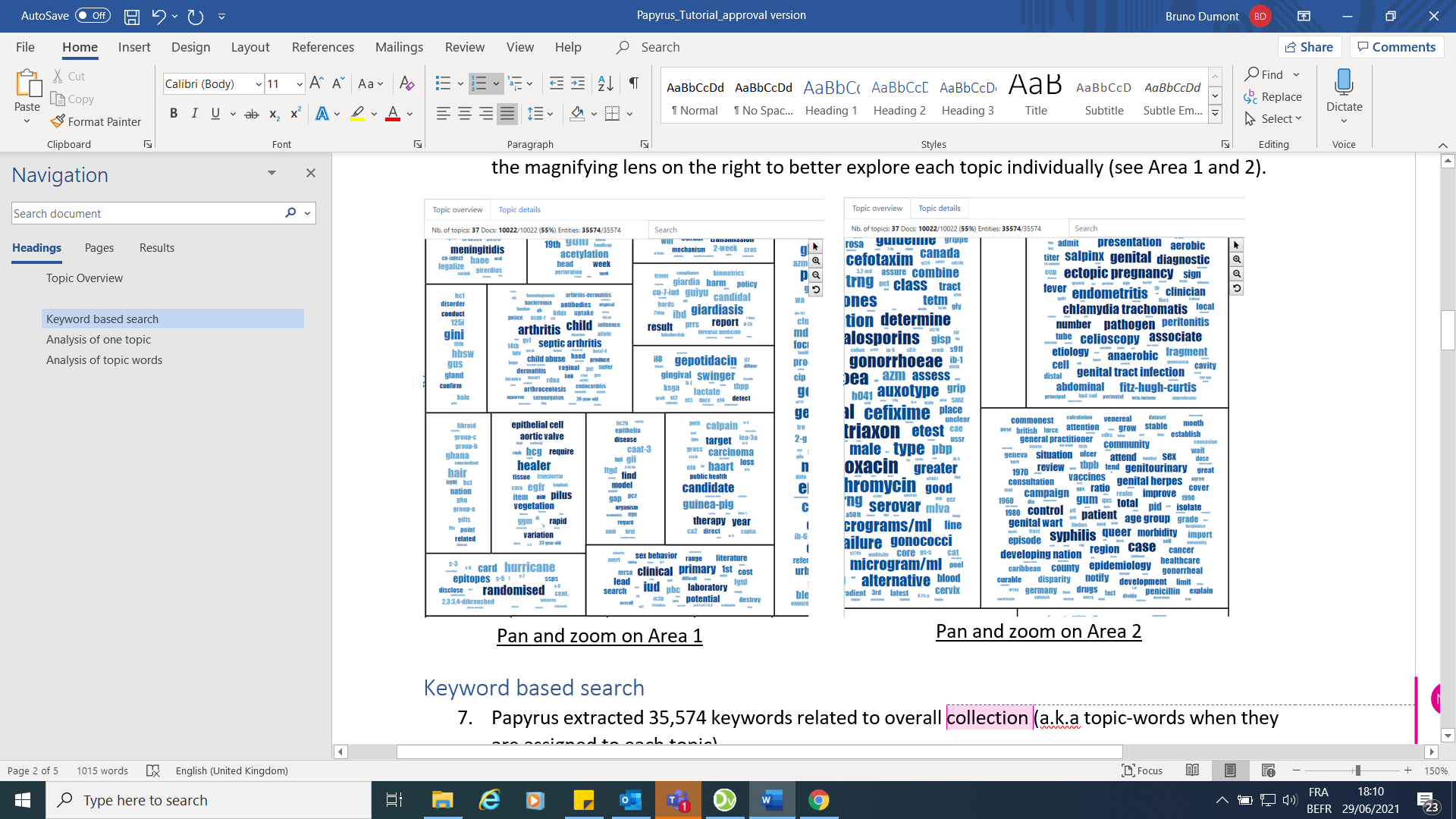


## Keyword based search

1. Papyrus extracted 35,574 keywords related to overall collection (also referred to as ‘topic-words’ when they are assigned to each topic).
2. Papyrus also supports keyword-based search through the search bar at the top of the map.
3. The topics matching the query are outlined in red, in this case in a search for ‘ectopic pregnancy’, a list of related topics is highlighted.


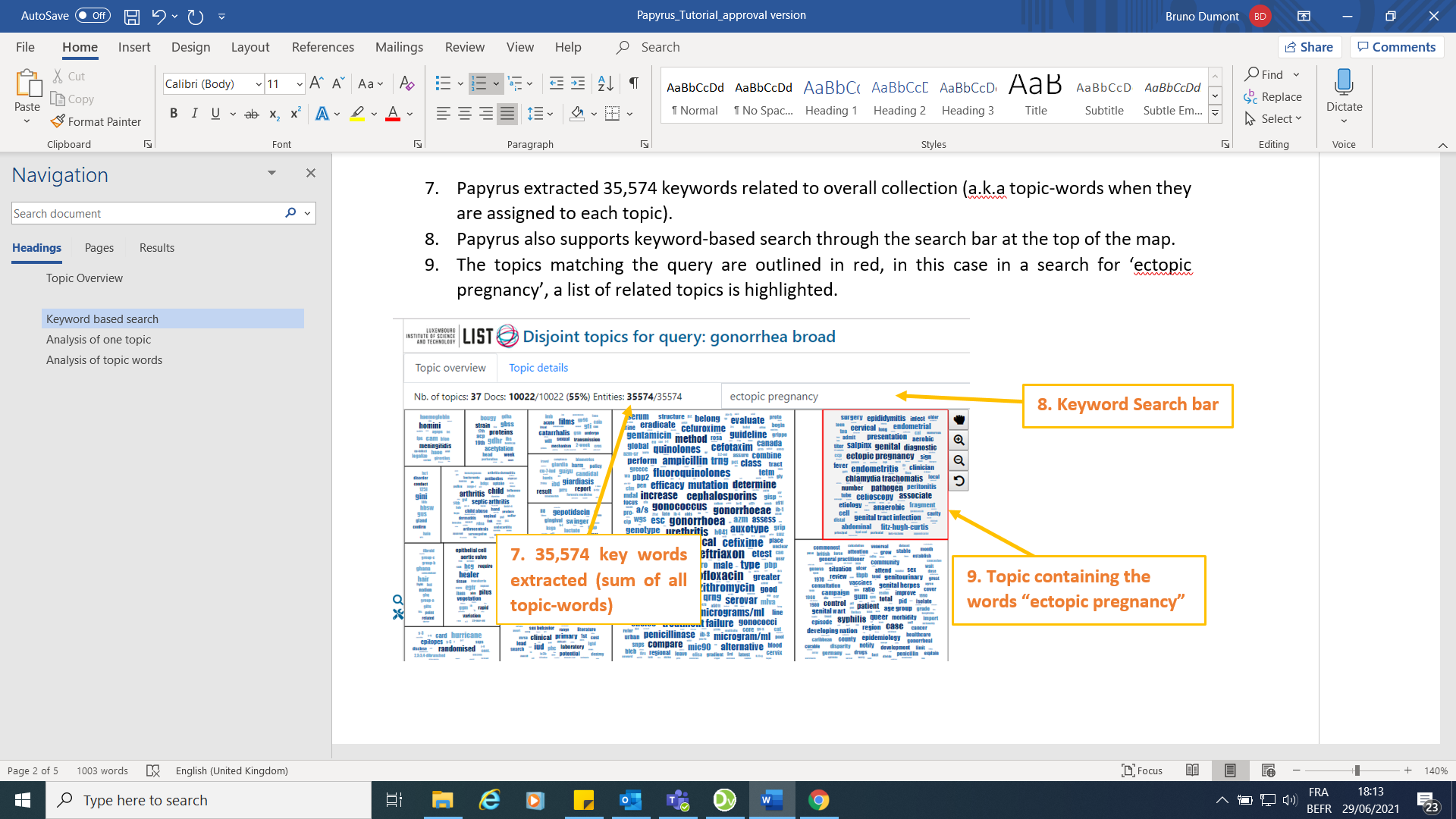


## Analysis of one topic

1. Placing the cursor over the tile will result in a report of metrics from the individual tile (e.g. 462 topic-words related to 591 documents). When the user clicks on a topic tile, the list of topic-words is displayed as a bar chart on the right side of the map. (Please note: if the bar chart is not visible, it is because the screen is too small. Use the scroll bars on the bottom of the screen or click on the ‘documents’ tab (
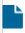
) on the far right to toggle between the bar chart and the list of related titles). The chart is sorted in descending order of word relevance for the topic. For example, in the tile on the top right (related to reproductive tract sequelae and containing the topic-words ‘ectopic pregnancy’), the relative frequency of 462 topic-words in the tile is presented, ectopic pregnancy occurring most frequently, followed by ‘genital’, ‘endometriosis’ etc.


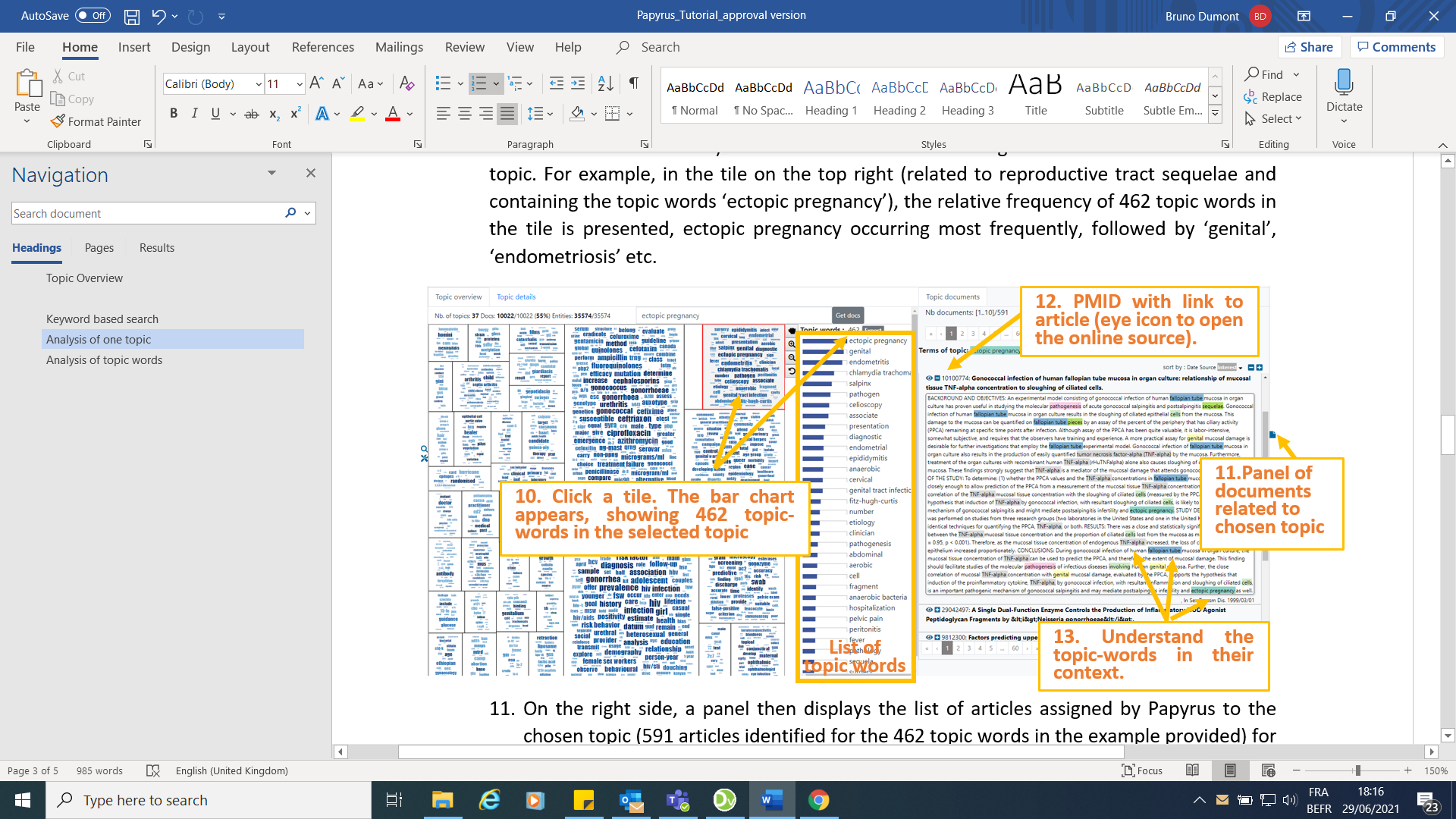


1. On the right side, a panel then displays the list of articles assigned by Papyrus to the chosen topic (591 articles identified for the 462 topic-words in the example provided) for the purpose of close reading. The list can be browsed through using the scroll bar and also page by page.

1. The PubMed ID (PMID) is provided along with an online link (click on the ‘eye’ icon) to the article for direct access in an internet browser.

1. By clicking on the ‘+’ icon next to a title, the textual content of the article is displayed, and the topic-words are highlighted in distinct colours and their exact meaning can be understood in their precise context.
2. Finally, the list of topic-words, related PubMed IDs, article titles and URLs for each topic can be exported in a CSV file (by clicking the export button in the configuration panel
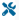
), for further scrutiny in third-party software. To address the research question addressed here, the export function was used to extract the full list of topic-words (an extract of which is presented in supplementary figure 1). The CSV file for each topic may contain duplicates of the same topic-word as the topic-word may have been extracted from multiple articles (e.g., for topic 36, the topic-word 'giardiasis' is listed 9 times as it was found in 9 articles). Using the duplication function of MS Excel, the final full list of unique topic-words (n=10,091, as reported in figure 3 in the paper) was then derived (e.g., considering only once the topic-word 'giardiasis'). The final topic-word list was then reviewed manually and relevant topic-words and related titles retained, as reported in the paper (please refer to Search 3 in the text for details).


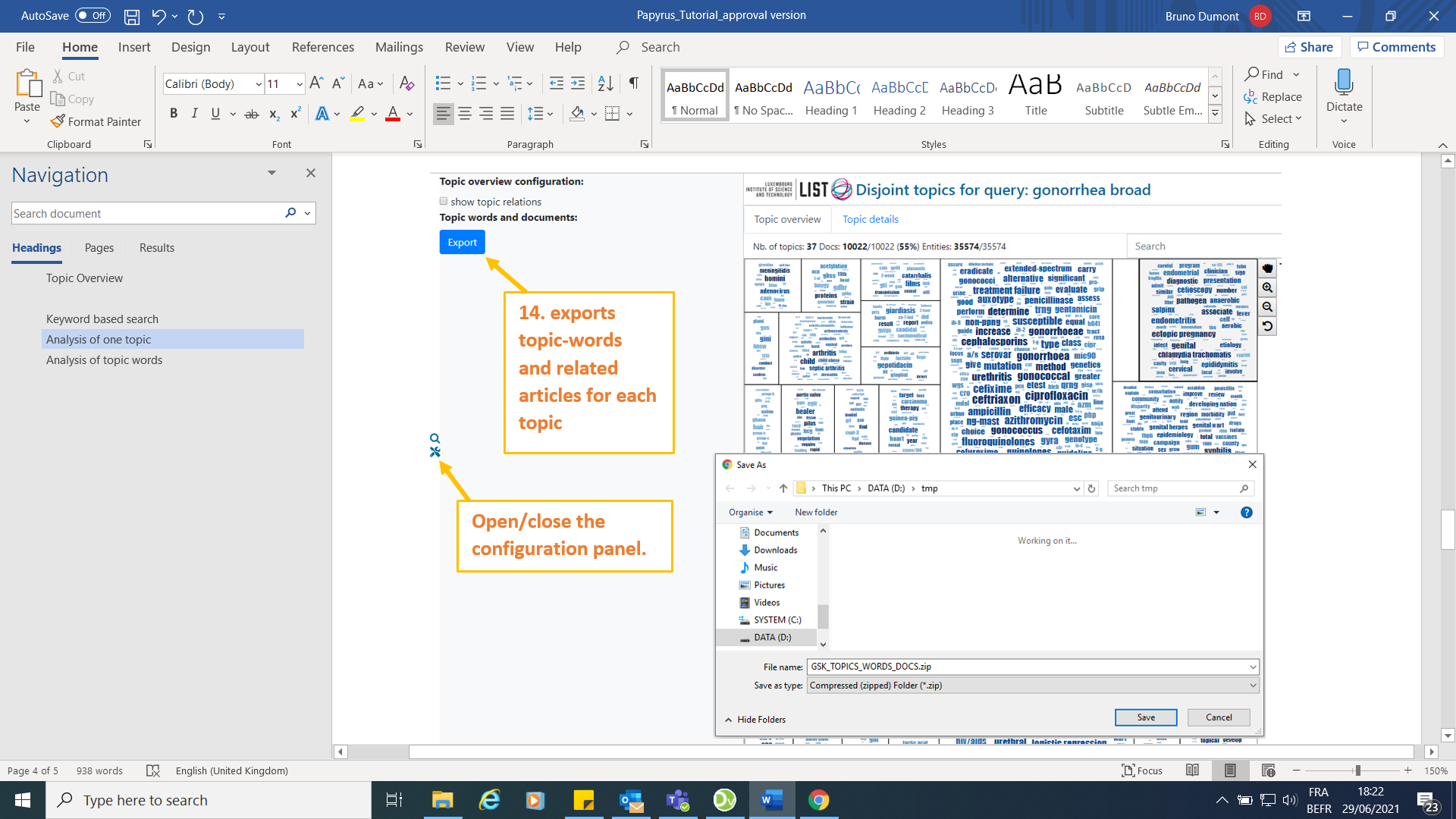


## Analysis of topic-words

In addition, by clicking on a topic-word of interest in the bar chart, the list of articles is filtered to only show the subset of articles containing this word.

If the topic-words are nouns or verbs, they are conflated into their canonical form by the Natural Language Processing process of Papyrus (in the lemmatization step). Likewise, topic-words corresponding to medical concepts (e.g. synonymous terms of the MeSH ontology) or geographical locations (e.g. Geonames) are conflated into their preferred form. When any of these concept forms is found in the text, they get the same highlight color for consistency (e.g. “tnf-alpha” and “tumor necrosis factor-alpha” highlighted in grey).


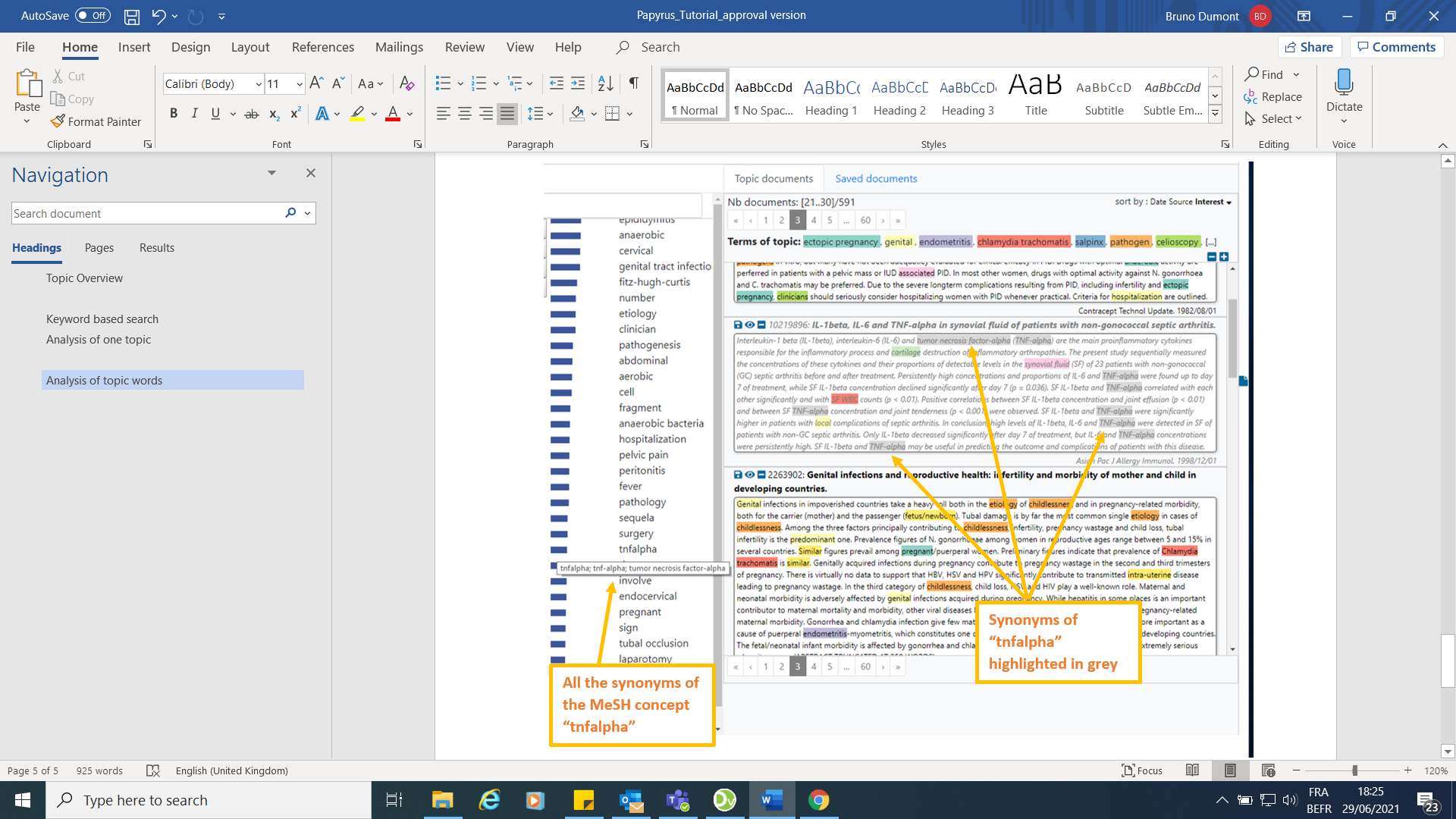

Supplement: Supplementary file 1 — Additional file 1: Supplementary Text 1. Papyrus tutorial. [file 12874_2021_1367_MOESM1_ESM.docx]
