# Supplementary material for: Applying a novel approach to scoping review incorporating artificial intelligence: mapping the natural history of gonorrhoea
Source: BMC Med Res Methodol. 2021 Sep 6;21:183. doi: 10.1186/s12874-021-01367-x (PMC8418964; doi:10.1186/s12874-021-01367-x)
Supplement: Supplementary file 2 — Additional file 2: Supplemental Fig. 1. Raw textual output listing a sample of representative words of one of the topics identified by the AI methods of the Papyrus tool from the corpus resulting from the search query ‘gonorrhea’. In this example, the most important topic-word (most frequently occurring) is ‘ectopic pregnancy’ followed by other words like ‘chlamydia trachomatis’ and ‘salpinx’. Supplementary Text 2.1. Public Health Websites accessed for the initial ‘high yield’ search. Supplementary Text 2.2. Seminal literature based on a review of key authors in the field. Supplementary Table 1. ICD9/ICD10 and Read (CPRD) codes. Supplementary Table 2. Read codes (level 3). [file 12874_2021_1367_MOESM2_ESM.docx]

# Additional File 2

##

## Supplementary figure 1. Raw textual output^¥^ listing a sample of representative words of one of the topics identified by the AI methods of the Papyrus tool from the corpus resulting from the search query ‘gonorrhea’. In this example, the most important topic-word (most frequently occurring) is ‘ectopic pregnancy’ followed by other words like ‘chlamydia trachomatis’ and ‘salpinx’


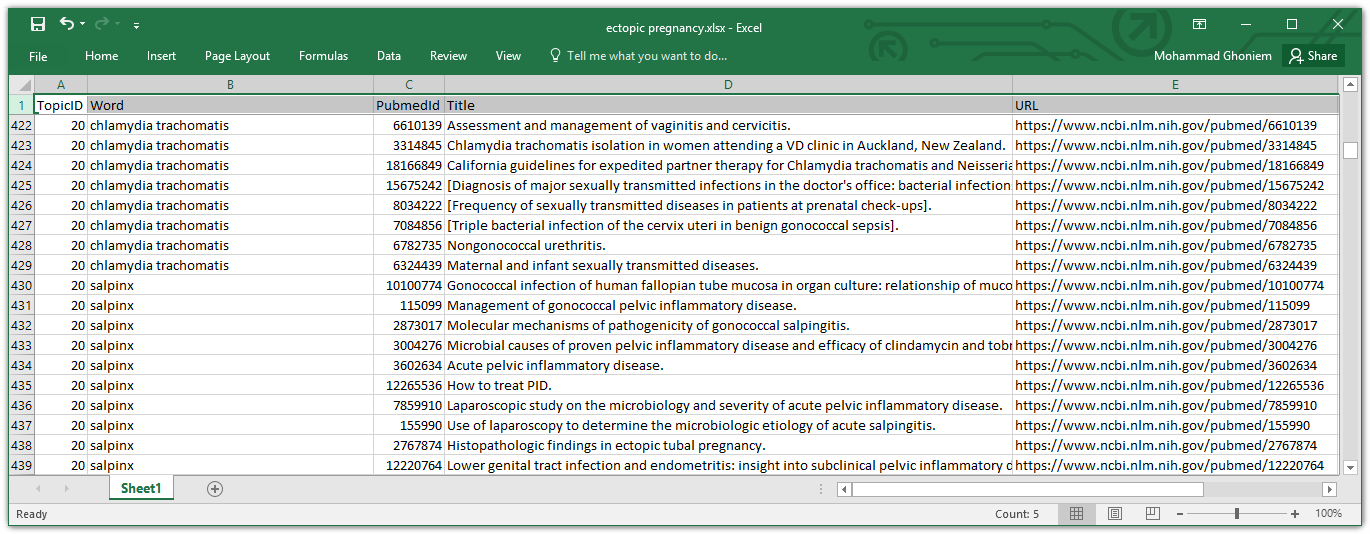


AI, artificial intelligence

^¥^View of raw data used for the manual screening of the words that are representative of the topic extracted by the AI methods of the Papyrus tool. For each topic extracted by the tool, a file similar to the one shown above was screened. Each row associates a representative topic-word of the topic at hand to the title and reference of an abstract where it appears. As a topic-word may belong to several abstracts within the same topic, the same word may be repeated in several row.

## Supplementary text 2. Lists of seminal papers, public health websites and ICD10/ICD9 codes

### Supplementary text 2.1. Public Health Websites accessed for initial ‘high yield’ search

- CDC

<https://www.cdc.gov/std/gonorrhea/stdfact-gonorrhea-detailed.htm>

- Robert Koch Institute

<https://www.rki.de/DE/Content/Infekt/EpidBull/Merkblaetter/Ratgeber_Gonorrhoe.html>

- National Health Services (England)

<https://www.nhs.uk/conditions/gonorrhoea/>

- British Association for Sexual Health and HIV (BASHH):

<https://www.bashhguidelines.org/media/1129/gonorrhoea-screen.pdf>

- National Institute of Public Health (RIVM), Netherlands:

<https://lci.rivm.nl/richtlijnen/gonorroe>

ICD 9/10, International Statistical Classification of Diseases and Related Health Problems (Ninth or Tenth Revision)

### Supplementary text 2.2. Seminal literature based on review of key authors in the field

- Bro-Jorgensen A, Jensen T. Gonococcal pharyngeal infections. Brit J Vener Dis 1973: 49, 491-9
- Cates W, Jr., Joesoef MR, Goldman MB. Atypical pelvic inflammatory disease: can we identify clinical predictors? American journal of obstetrics and gynecology. 1993;169(2 Pt 1):341-6.
- Edwards & Apicella, 2004: The Molecular Mechanisms Used by Neisseria gonorrhoeae To Initiate Infection Differ between Men and Women
- Edwards & Butler, 2011: The Pathobiology of Neisseria gonorrhoeae lower female genital tract
- Edwards JL et al. 2016: Is gonococcal disease preventable? The importance of understanding immunity and pathogenesis in vaccine development.
- Grodstein F, Goldman MB, Cramer DW. Relation of tubal infertility to history of sexually transmitted diseases. American journal of epidemiology. 1993;137(5):577-84.
- Lovett & Duncan, 2018: Human immmune responses and the natural history of Neisseria gonorrhoeae infection.
- Miller, KE. Diagnosis and Treatment of Neisseria gonorrhoeae Infections*Am Fam Physician.* 2006 May 15;73(10):1779-1784.
- Ndowa 2012: The threat of untreatable gonorrhoea: implications and consequences for reproductive and sexual morbidity:, <https://www.tandfonline.com/doi/full/10.1016/S0968-8080(12)40653-X>
- Quillin 2018. Neisseria gonorrhoeae host adaptation and pathogenesis. Nat Rev Micriobiol 16 (4), 226-240
- Reekie J, Roberts C, Preen D, Hocking JS, Donovan B, Ward J, et al. Chlamydia trachomatis and the risk of spontaneous preterm birth, babies who are born small for gestational age, and stillbirth: a population-based cohort study. The Lancet Infectious diseases. 2018;18(4):452-60.
- Sherman KJ, Daling JR, Weiss NS. Sexually transmitted diseases and tubal infertility. Sexually transmitted diseases. 1987;14(1):12-6.

### Supplementary table 1. ICD9/ICD10 and Read (CPRD) codes

**ICD9/ICD10**

| Gonococcal Infections | 098 | ICD9 |
| --- | --- | --- |
| Gonococcal Infection (Acute) Of Lower Genitourinary Tract | 0980 | ICD9 |
| Gonococcal Infection (Acute) Of Upper Genitourinary Tract | 0981 | ICD9 |
| Gonococcal Infection (Acute) Of Upper Genitourinary Tract Site Unspecified | 09810 | ICD9 |
| Gonococcal Cystitis (Acute) | 09811 | ICD9 |
| Gonococcal Prostatitis (Acute) | 09812 | ICD9 |
| Gonococcal Epididymo-Orchitis (Acute) | 09813 | ICD9 |
| Gonococcal Seminal Vesiculitis (Acute) | 09814 | ICD9 |
| Gonococcal Cervicitis (Acute) | 09815 | ICD9 |
| Gonococcal Endometritis (Acute) | 09816 | ICD9 |
| Gonococcal Salpingitis Specified As Acute | 09817 | ICD9 |
| Other Gonococcal Infection (Acute) Of Upper Genitourinary Tract | 09819 | ICD9 |
| Gonococcal Infection Chronic Of Lower Genitourinary Tract | 0982 | ICD9 |
| Gonococcal Infection Chronic Of Upper Genitourinary Tract | 0983 | ICD9 |
| Chronic Gonococcal Infection Of Upper Genitourinary Tract Site Unspecified | 09830 | ICD9 |
| Gonococcal Cystitis Chronic | 09831 | ICD9 |
| Gonococcal Prostatitis Chronic | 09832 | ICD9 |
| Gonococcal Epididymo-Orchitis Chronic | 09833 | ICD9 |
| Gonococcal Seminal Vesiculitis Chronic | 09834 | ICD9 |
| Gonococcal Cervicitis Chronic | 09835 | ICD9 |
| Gonococcal Endometritis Chronic | 09836 | ICD9 |
| Gonococcal Salpingitis (Chronic) | 09837 | ICD9 |
| Other Chronic Gonococcal Infection Of Upper Genitourinary Tract | 09839 | ICD9 |
| Gonococcal Infection Of Eye | 0984 | ICD9 |
| Gonococcal Conjunctivitis (Neonatorum) | 09840 | ICD9 |
| Gonococcal Iridocyclitis | 09841 | ICD9 |
| Gonococcal Endophthalmia | 09842 | ICD9 |
| Gonococcal Keratitis | 09843 | ICD9 |
| Other Gonococcal Infection Of Eye | 09849 | ICD9 |
| Gonococcal Infection Of Joint | 0985 | ICD9 |
| Gonococcal Arthritis | 09850 | ICD9 |
| Gonococcal Synovitis And Tenosynovitis | 09851 | ICD9 |
| Gonococcal Bursitis | 09852 | ICD9 |
| Gonococcal Spondylitis | 09853 | ICD9 |
| Other Gonococcal Infection Of Joint | 09859 | ICD9 |
| Gonococcal Infection Of Pharynx | 0986 | ICD9 |
| Gonococcal Infection Of Anus And Rectum | 0987 | ICD9 |
| Gonococcal Infection Of Other Specified Sites | 0988 | ICD9 |
| Gonococcal Keratosis (Blennorrhagica) | 09881 | ICD9 |
| Gonococcal Meningitis | 09882 | ICD9 |
| Gonococcal Pericarditis | 09883 | ICD9 |
| Gonococcal Endocarditis | 09884 | ICD9 |
| Other Gonococcal Heart Disease | 09885 | ICD9 |
| Gonococcal Peritonitis | 09886 | ICD9 |
| Gonococcal Infection Of Other Specified Sites | 09889 | ICD9 |
| Other Nongonococcal Urethritis | 0994 | ICD9 |
| Other Nongonococcal Urethritis Unspecified | 09940 | ICD9 |
| Other Nongonococcal Urethritis Chlamydia Trachomatis | 09941 | ICD9 |
| Other Nongonococcal Urethritis Other Specified Organism | 09949 | ICD9 |
| Gonorrhea Complicating Pregnancy Childbirth Or The Puerperium | 6471 | ICD9 |
| Gonorrhea Of Mother Complicating Pregnancy Childbirth Or The Puerperium Unspecified As To Episode Of Care | 64710 | ICD9 |
| Gonorrhea Of Mother With Delivery | 64711 | ICD9 |
| Gonorrhea Of Mother With Delivery With Postpartum Complication | 64712 | ICD9 |
| Antepartum Gonorrhea | 64713 | ICD9 |
| Postpartum Gonorrhea | 64714 | ICD9 |
| Gonococcal Infection | A54 | ICD10 |
| Gonococcal Infection Of Lower Genitourinary Tract Without Periurethral Or Accessory Gland Abscess | A540 | ICD10 |
| Gonococcal Infection Of Lower Genitourinary Tract, Unspecified | A5400 | ICD10 |
| Gonococcal Cystitis And Urethritis, Unspecified | A5401 | ICD10 |
| Gonococcal Vulvovaginitis, Unspecified | A5402 | ICD10 |
| Gonococcal Cervicitis, Unspecified | A5403 | ICD10 |
| Other Gonococcal Infection Of Lower Genitourinary Tract | A5409 | ICD10 |
| Gonococcal Infection Of Lower Genitourinary Tract With Periurethral And Accessory Gland Abscess | A541 | ICD10 |
| Gonococcal Pelviperitonitis And Other Gonococcal Genitourinary Infection | A542 | ICD10 |
| Gonococcal Infection Of Kidney And Ureter | A5421 | ICD10 |
| Gonococcal Prostatitis | A5422 | ICD10 |
| Gonococcal Infection Of Other Male Genital Organs | A5423 | ICD10 |
| Gonococcal Female Pelvic Inflammatory Disease | A5424 | ICD10 |
| Other Gonococcal Genitourinary Infections | A5429 | ICD10 |
| Gonococcal Infection Of Eye | A543 | ICD10 |
| Gonococcal Infection Of Eye, Unspecified | A5430 | ICD10 |
| Gonococcal Conjunctivitis | A5431 | ICD10 |
| Gonococcal Iridocyclitis | A5432 | ICD10 |
| Gonococcal Keratitis | A5433 | ICD10 |
| Other Gonococcal Eye Infection | A5439 | ICD10 |
| Gonococcal Infection Of Musculoskeletal System | A544 | ICD10 |
| Gonococcal Infection Of Musculoskeletal System, Unspecified | A5440 | ICD10 |
| Gonococcal Spondylopathy | A5441 | ICD10 |
| Gonococcal Arthritis | A5442 | ICD10 |
| Gonococcal Osteomyelitis | A5443 | ICD10 |
| Gonococcal Infection Of Other Musculoskeletal Tissue | A5449 | ICD10 |
| Gonococcal Pharyngitis | A545 | ICD10 |
| Gonococcal Infection Of Anus And Rectum | A546 | ICD10 |
| Other Gonococcal Infections | A548 | ICD10 |
| Gonococcal Meningitis | A5481 | ICD10 |
| Gonococcal Brain Abscess | A5482 | ICD10 |
| Gonococcal Heart Infection | A5483 | ICD10 |
| Gonococcal Pneumonia | A5484 | ICD10 |
| Gonococcal Peritonitis | A5485 | ICD10 |
| Gonococcal Sepsis | A5486 | ICD10 |
| Other Gonococcal Infections | A5489 | ICD10 |
| Gonococcal Infection, Unspecified | A549 | ICD10 |
| Gonorrhea Complicating Pregnancy, Childbirth And The Puerperium | O982 | ICD10 |
| Gonorrhea Complicating Pregnancy | O9821 | ICD10 |
| Gonorrhea Complicating Pregnancy, First Trimester | O98211 | ICD10 |
| Gonorrhea Complicating Pregnancy, Second Trimester | O98212 | ICD10 |
| Gonorrhea Complicating Pregnancy, Third Trimester | O98213 | ICD10 |
| Gonorrhea Complicating Pregnancy, Unspecified Trimester | O98219 | ICD10 |
| Gonorrhea Complicating Childbirth | O9822 | ICD10 |
| Gonorrhea Complicating The Puerperium | O9823 | ICD10 |
| Carrier Or Suspected Carrier Of Gonorrhea | V027 | ICD9 |

ICD 9/10, International Statistical Classification of Diseases and Related Health Problems (Ninth or Tenth Revision); CPRD, Clinical Practice Research Datalink

### Supplementary table 2. Read codes (level 3)

| 1233.00 | 94269 | FH: Gonorrhoea |
| --- | --- | --- |
| 1415100 | 102683 | H/O: gonorrhoea |
| 43E6.00 | 42020 | Gonorrhoea infect. titre test |
| 43E6.11 | 32935 | Gonorrhoea titre test |
| 43h6.00 | 27182 | Neisseria gonorrhoeae polymerase chain reaction |
| 43jA.00 | 49013 | Neisseria gonorrhoeae nucleic acid detection |
| 43jA000 | 108619 | Neisseria gonorrhoeae nucleic acid detection assay |
| 4J2D.00 | 109749 | Neisseria gonorrhoeae culture |
| 4JF4300 | 104460 | Throat swab for gonorrhoea |
| 4JH6400 | 104462 | Anal swab for gonorrhoea |
| 4JKB.00 | 32729 | Gonococcal cervical swab |
| 4JKC.00 | 29255 | Gonococcal urethral swab |
| 4JLA.00 | 50562 | Gonococcal swab |
| 4JQ8.00 | 67063 | Gonorrhoea test negative |
| 4JQ9.00 | 96812 | Gonorrhoea test equivocal |
| 4JQA.00 | 60038 | Gonorrhoea test positive |
| 65P7.11 | 31803 | Gonorrhoea contact |
| 65PK.00 | 53222 | Gonorrhoea contact |
| 65Q8.00 | 52628 | Gonorrhoea carrier |
| 677M.00 | 67177 | Gonorrhoea screening counselling |
| 8I3o.00 | 96166 | Gonorrhoea screening declined |
| 9kF2.00 | 96772 | Urine screen for gonorrhoea - enhanced services administratn |
| 9kF2.11 | 97115 | Urine screen for gonorrhoea |
| 9ka..00 | 96573 | Urine neisseria gonorrhoeae test positive - enhan serv admin |
| 9ka..11 | 100404 | Urine neisseria gonorrhoeae test positive |
| 9kb..00 | 98045 | Urine neisseria gonorrhoeae test negative - enhan serv admin |
| 9kb..11 | 103950 | Urine neisseria gonorrhoeae test negative |
| A98..00 | 3965 | Gonococcal infections |
| A980.00 | 23460 | Acute gonorrhoea of lower genitourinary tract |
| A980000 | 44279 | Acute gonococcal Bartholinitis |
| A980100 | 4788 | Acute gonococcal urethritis |
| A980200 | 1995 | Acute gonococcal vulvovaginitis |
| A980z00 | 40228 | Acute gonorrhoea of lower genitourinary tract NOS |
| A981.00 | 94523 | Acute gonorrhoea of upper genitourinary tract |
| A981000 | 112011 | Acute unspecified gonorrhoea of upper genitourinary tract |
| A981100 | 10295 | Acute gonococcal cystitis |
| A981111 | 97403 | Bladder gonorrhoea - acute |
| A981200 | 17966 | Acute gonococcal prostatitis |
| A981300 | 20018 | Acute gonococcal epididymo-orchitis |
| A981311 | 48100 | Acute gonococcal orchitis |
| A981400 | 96427 | Acute gonococcal seminal vesiculitis |
| A981500 | 31366 | Acute gonococcal cervicitis |
| A981600 | 8716 | Acute gonococcal endometritis |
| A981611 | 97359 | Uterus - acute gonorrhoea |
| A981700 | 31380 | Acute gonococcal salpingitis |
| A981z00 | 61131 | Acute gonorrhoea upper genitourinary tract NOS |
| A982.00 | 59489 | Chronic gonorrhoea lower genitourinary tract |
| A982000 | 94520 | Chronic gonococcal bartholinitis |
| A982100 | 12018 | Chronic gonococcal urethritis |
| A982200 | 69423 | Chronic gonococcal vulvovaginitis |
| A982z00 | 106493 | Chronic gonorrhoea of lower genitourinary tract NOS |
| A983.00 | 97383 | Chronic gonorrhoea of upper genitourinary tract |
| A983000 | 112012 | Chronic unspecified gonorrhoea of upper genitourinary tract |
| A983100 | 48908 | Chronic gonococcal cystitis |
| A983200 | 19237 | Chronic gonococcal prostatitis |
| A983300 | 50542 | Chronic gonococcal epididymo-orchitis |
| A983400 | 112013 | Chronic gonococcal seminal vesiculitis |
| A983500 | 17337 | Chronic gonococcal cervicitis |
| A983600 | 51488 | Chronic gonococcal endometritis |
| A983611 | 112014 | Uterus - chronic gonorrhoea |
| A983700 | 16101 | Chronic gonococcal salpingitis |
| A983z00 | 112015 | Chronic gonorrhoea of upper genitourinary tract NOS |
| A984.00 | 17599 | Gonococcal eye infection |
| A984000 | 44062 | Neonatal gonococcal conjunctivits |
| A984011 | 24239 | Ophthalmia neonatorum - gonococcal |
| A984100 | 106506 | Gonococcal iridocyclitis |
| A984200 | 62190 | Gonococcal endophthalmia |
| A984300 | 72172 | Gonococcal keratitis |
| A984z00 | 5064 | Gonococcal eye infection NOS |
| A985.00 | 57670 | Gonococcal joint infection |
| A985000 | 23443 | Gonococcal arthritis |
| A985100 | 49377 | Gonococcal synovitis or tenosynovitis |
| A985111 | 60087 | Gonococcal synovitis |
| A985112 | 43481 | Gonococcal tenosynovitis |
| A985200 | 42420 | Gonococcal bursitis |
| A985300 | 51894 | Gonococcal spondylitis |
| A985z00 | 104978 | Gonococcal joint infection NOS |
| A985z11 | 67955 | Rheumatism - gonococcal |
| A986.00 | 50882 | Gonococcal pharynx infection |
| A987.00 | 47200 | Gonococcal proctitis |
| A987000 | 67564 | Gonococcal anal infection |
| A987100 | 52375 | Gonococcal rectal infection |
| A987z00 | 73699 | Gonococcal proctitis NOS |
| A98y.00 | 71967 | Gonococcal infection of other specified sites |
| A98y000 | 56496 | Gonococcal keratosis |
| A98y100 | 112016 | Gonococcal meningitis |
| A98y200 | 96449 | Gonococcal pericarditis |
| A98y300 | 33364 | Gonococcal endocarditis |
| A98y400 | 112017 | Other gonococcal heart disease |
| A98y500 | 39736 | Gonococcal peritonitis |
| A98yy00 | 40219 | Other gonococcal infection of other specified site |
| A98yy11 | 71114 | Gonococcal hepatitis |
| A98yy12 | 46100 | Abscess gonococcal |
| A98yy13 | 93945 | Gonococcal perihepatitis |
| A98yy14 | 4126 | Gonococcal cellulitis |
| A98yz00 | 99680 | Gonococcal infection of other site NOS |
| A98yz11 | 54561 | Gonococcaemia NOS |
| A98yz12 | 5104 | Gonococcal septicaemia |
| A98z.00 | 37545 | Gonococcal infections NOS |
| A98z.11 | 274 | Gonorrhoea |
| Ayu4B00 | 112025 | [X]Other gonococcal infections |
| Ayu4C00 | 73252 | [X]Gonococcal infection, unspecified |
| F007000 | 112039 | Meningitis due to gonococcus |
| G500500 | 103850 | Acute pericarditis - gonococcal |
| G511200 | 100572 | Endocarditis - gonococcal |
| J550000 | 70431 | Peritonitis - gonococcal |
| K154500 | 68954 | Cystitis in gonorrhoea |
| K214400 | 70508 | Prostatitis in gonorrhoea |
| K44..00 | 10264 | Female gonococcal pelvic inflammatory disease |
| L171.00 | 41961 | Maternal gonorrhoea during pregnancy/childbirth/puerperium |
| L171000 | 112124 | Maternal gonorrhoea, unspec whether in pregnancy/puerperium |
| L171100 | 112125 | Maternal gonorrhoea during pregnancy - baby delivered |
| L171200 | 112126 | Maternal gonorrhoea in puerperium - baby delivered |
| L171300 | 41959 | Maternal gonorrhoea in pregnancy - baby not yet delivered |
| L171400 | 112127 | Maternal gonorrhoea in puerperium- baby previously delivered |
| L171z00 | 112128 | Maternal gonorrhoea in pregnancy/childbirth/puerperium NOS |
| ZV02700 | 95061 | [V]Gonorrhoea carrier |
