# Supplementary material for: Applying a novel approach to scoping review incorporating artificial intelligence: mapping the natural history of gonorrhoea
Source: BMC Med Res Methodol. 2021 Sep 6;21:183. doi: 10.1186/s12874-021-01367-x (PMC8418964; doi:10.1186/s12874-021-01367-x)
Supplement: Supplementary file 3 — Additional file 3: Supplementary Table 3 Systematic search Strategy and number of records retrieved. Supplementary Table 4 Inclusions from systematic search. [file 12874_2021_1367_MOESM3_ESM.docx]

## Additional file 3. Systematic search strategy and article selection

### Supplementary table 3. Systematic search Strategy and number of records retrieved

| **Search No.** | **Search Strategy** | | **4-11-2019** | |
| --- | --- | --- | --- | --- |
|  | **Category** | **Terms** | **Pubmed Search** | **No. records retrieved** |
| **1** | Key Words: Combined with 'OR' | gonorr*[Title/Abstract] | (((gonorr*[Title/Abstract] OR "neisseria gonorrhoeae"[Title/Abstract] OR Ng[Title/Abstract] OR gonococc*[Title/Abstract] OR  N. gonorrh*))[Title/Abstract]) | **n=242770** |
|  |  | "neisseria gonorrhoeae"[Title/Abstract] |  |  |
|  |  | Ng[Title/Abstract] |  |  |
|  |  | N. gonorrh*[Title/Abstract]) |  |  |
|  |  | gonococc*[Title/Abstract] |  |  |
| **2** | Key Words: Combined with 'OR" | 'natural history' | ((('natural history'[Title/Abstract] OR pathogenesis[Title/Abstract] OR pathobiology[Title/Abstract] OR lifecycle[Title/Abstract] OR  'disease progression'[Title/Abstract])) | **n=431772** |
|  |  | pathogenesis |  |  |
|  |  | pathobiology |  |  |
|  |  | lifecycle |  |  |
|  |  | disease progression' |  |  |
| **3** | Outcome: Search 1 and 2 | Combined with "AND" | ((('natural history'[Title/Abstract] OR pathogenesis[Title/Abstract] OR pathobiology[Title/Abstract] OR lifecycle[Title/Abstract] OR  'disease progression'[Title/Abstract]))) AND ((gonorr*[Title/Abstract] OR "neisseria gonorrhoeae"[Title/Abstract] OR Ng[Title/Abstract] OR gonococc*[Title/Abstract] OR N. gonorrh*[Title/Abstract]))) | **n=4448** |
| **4** | Comparison (MeSH)  Combined with 'OR' | "Neisseria gonorrhoeae/genetics"[Majr] | (( "Neisseria gonorrhoeae/genetics"[Majr] OR  "Neisseria gonorrhoeae/growth and development"[Majr] OR  "Neisseria gonorrhoeae/immunology"[Majr] OR  "Neisseria gonorrhoeae/metabolism"[Majr] OR  "Neisseria gonorrhoeae/pathogenicity"[Majr] OR  "Neisseria gonorrhoeae/physiology"[Majr] )) OR  ( "Gonorrhea/genetics"[Majr] OR  "Gonorrhea/immunology"[Majr] OR  "Gonorrhea/pathology"[Majr] OR  "Gonorrhea/physiology"[Majr] OR  "Gonorrhea/physiopathology"[Majr] ) | **n=3343** |
|  |  | "Neisseria gonorrhoeae/growth and development"[Majr] |  |  |
|  |  | "Neisseria gonorrhoeae/immunology"[Majr] |  |  |
|  |  | "Neisseria gonorrhoeae/metabolism"[Majr] |  |  |
|  |  | "Neisseria gonorrhoeae/pathogenicity"[Majr] |  |  |
|  |  | "Neisseria gonorrhoeae/physiology"[Majr] |  |  |
|  |  | "Gonorrhea/genetics"[Majr] |  |  |
|  |  | "Gonorrhea/immunology"[Majr] |  |  |
|  |  | "Gonorrhea/pathology"[Majr] |  |  |
|  |  | "Gonorrhea/physiology"[Majr] |  |  |
|  |  | "Gonorrhea/physiopathology"[Majr] |  |  |
| **7** | Search 3 and 4 | Combined with 'AND' | ((((( "Neisseria gonorrhoeae/genetics"[Majr] OR  "Neisseria gonorrhoeae/growth and development"[Majr] OR  "Neisseria gonorrhoeae/immunology"[Majr] OR  "Neisseria gonorrhoeae/metabolism"[Majr] OR  "Neisseria gonorrhoeae/pathogenicity"[Majr] OR  "Neisseria gonorrhoeae/physiology"[Majr] )) OR  ( "Gonorrhea/genetics"[Majr] OR "Gonorrhea/immunology"[Majr] OR "Gonorrhea/pathology"[Majr] OR "Gonorrhea/physiology"[Majr] OR "Gonorrhea/physiopathology"[Majr] )))) AND (((('natural history'[Title/Abstract] OR pathogenesis[Title/Abstract] OR pathobiology[Title/Abstract] OR lifecycle[Title/Abstract] OR  'disease progression'[Title/Abstract]))) AND ((gonorr*[Title/Abstract] OR "neisseria gonorrhoeae"[Title/Abstract] OR Ng[Title/Abstract] OR gonococc*[Title/Abstract] OR N. gonorrh*[Title/Abstract])))) | **n=145** |
| **8** | Limits | Humans |  | **n=101** |

### Supplementary table 4: Inclusions from systematic search

| **PMID** | **Author** | **Year of publication** | **Title** | **Reference** |
| --- | --- | --- | --- | --- |
| 31119633 | Laniewski et al. | 2019 | Analysis of Host Responses to Neisseria gonorrhoeae Using a Human Three-Dimensional Endometrial Epithelial Cell Model. | Methods Mol Biol. 2019;1997:347-361. doi: 10.1007/978-1-4939-9496-0_20. |
| 31119630 | Almonacid-Mendoza et al. | 2019 | Basic Methods for Examining Neisseria gonorrhoeae Interactions with Host Cells In Vitro. | Methods Mol Biol. 2019;1997:281-299. doi: 10.1007/978-1-4939-9496-0_17. |
| 15489357 | Edwards et al. | 2004 | The Molecular Mechanisms Used by Neisseria Gonorrhoeae to Initiate Infection Differ Between Men and Women | Clin Microbiol Rev. 2004 Oct;17(4):965-81, table of contents. Review. |
| 11553013 | Edwards et al. | 2004 | The Role of Complement Receptor 3 (CR3) in Neisseria Gonorrhoeae Infection of Human Cervical Epithelia | Cell Microbiol. 2001 Sep;3(9):611-22. |
| 26805040 | Edwards et al. | 2016 | Is gonococcal disease preventable? The importance of understanding immunity and pathogenesis in vaccine development. | Crit Rev Microbiol. 2016 Nov;42(6):928-41. doi: 10.3109/1040841X.2015.1105782. Epub 2016 Jan 23. Review. |
| 19480997 | Garcí­a-De La Torre et al. | 2009 | Gonococcal and nongonococcal arthritis. | Rheum Dis Clin North Am. 2009 Feb;35(1):63-73. doi: 10.1016/j.rdc.2009.03.001. Review. |
| 30524442 | Lenz et al. | 2018 | Pathogenesis of Neisseria gonorrhoeae and the Host Defense in Ascending Infections of Human Fallopian Tube. | Front Immunol. 2018 Nov 21;9:2710. doi: 10.3389/fimmu.2018.02710. eCollection 2018. Review. |
| 153144 | Ludivico et al. | 1979 | Survey for Immune Complexes in Disseminated Gonococcal Arthritis-Dermatitis Syndrome | Arthritis Rheum. 1979 Jan;22(1):19-24. |
| 15963875 | Marrazzo et al. | 2005 | Mucopurulent Cervicitis: No Longer Ignored, but Still Misunderstood | Infect Dis Clin North Am. 2005 Jun;19(2):333-49, viii. Review. |
| 16714596 | Morales et al. | 2006 | Infection of human fallopian tube epithelial cells with Neisseria gonorrhoeae protects cells from tumor necrosis factor alpha-induced apoptosis. | Infect Immun. 2006 Jun;74(6):3643-50. |
| 29430011 | Quillin et al. | 2018 | Neisseria gonorrhoeae host adaptation and pathogenesis. | Nat Rev Microbiol. 2018 Apr;16(4):226-240. doi: 10.1038/nrmicro.2017.169. Epub 2018 Feb 12. Review. |
| 31119614 | Shaughnessy et al. | 2019 | Biology of the Gonococcus: Disease and Pathogenesis. | Methods Mol Biol. 2019;1997:1-27. doi: 10.1007/978-1-4939-9496-0_1. Review. |
| 15839892 | Timmerman et al. | 2005 | Ultrastructural Analysis of the Pathogenesis of Neisseria Gonorrhoeae Endometrial Infection | Cell Microbiol. 2005 May;7(5):627-36. |
| 16210106 | Woods et al. | 2005 | Gonococcal infections in neonates and young children. | Semin Pediatr Infect Dis. 2005 Oct;16(4):258-70. Review. |

PMID, Pubmed identification number
