# Supplementary material for: Applying a novel approach to scoping review incorporating artificial intelligence: mapping the natural history of gonorrhoea
Source: BMC Med Res Methodol. 2021 Sep 6;21:183. doi: 10.1186/s12874-021-01367-x (PMC8418964; doi:10.1186/s12874-021-01367-x)
Supplement: Supplementary file 4 — Additional file 4: Supplementary Text 4.1. Description of the Papyrus system. Supplementary text 4.2. Interactive visualization. Supplementary Text 4.3. Reproducibility and General Applicability of this Approach. [file 12874_2021_1367_MOESM4_ESM.docx]

## Additional file 4: Papyrus

Papyrus^[[1]](#footnote-1)^ is a visual text analytics software developed initially to support investigative journalists in the evidence gathering and verification work they carry out in the process of writing a paper. In this line of work, analysts seek to be exhaustive to make sure that they did not miss an interesting lead nor overlook an important aspect of the question they are addressing. At the same time, they also need to be able to trace all claims back to their sources. In essence, systematic literature review in the scientific community has similar traits. In the present paper, we describe the main characteristics of Papyrus, its text preprocessing pipeline, and its main graphical user interface. We also reflect on the reproducibility of the results obtained by this approach and its suitability for similar literature reviews.

### Supplementary text 4.1 Description of the Papyrus system

The main difficulty in analyzing textual data stems from its unstructured nature. The Papyrus tool relies on statistical natural language processing (NLP) technology to model the textual contents of large corpora and to extract automatically its latent topics, without assuming any prior knowledge.

The Text Preprocessing Pipeline


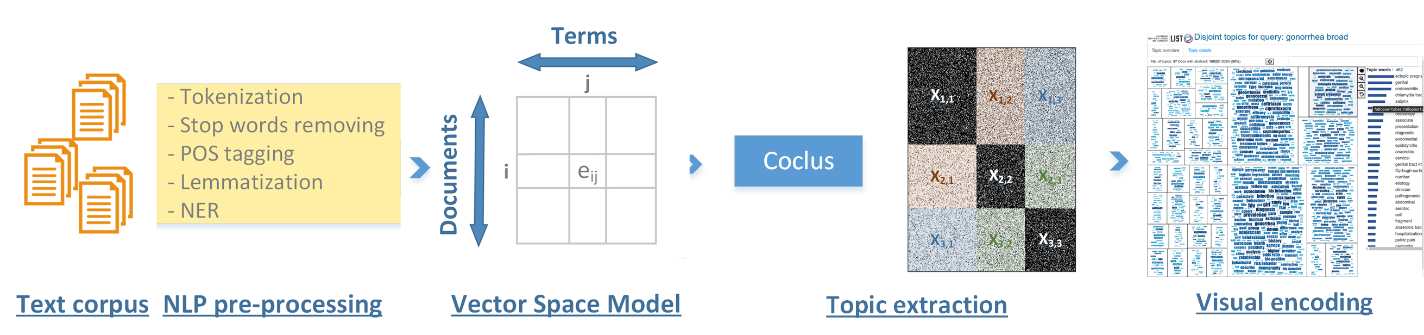


Supplementary figure 2. The general text processing pipeline of Papyrus comprises four steps: (1) NLP preprocessing, (2) vector space modeling, (3) topic extraction and (4) interactive visualization.

1. We use the Stanford Core NLP library^[[2]](#footnote-2)^ to achieve the following text preprocessing steps:

- Tokenization – In this step, each paper abstract is segmented into its individual words.
- Removal of ‘stop words’ – This corresponds to short function words, such as ‘the’, ‘is’, and ‘at’, which do not carry much semantic information, despite being very frequent in natural language.
- Lemmatization – In this step, words sharing the same dictionary entry with various inflectional endings are conflated into one entry (infects => infect; clinicians => clinician). (e.g. the plural and singular forms of the same word, like ‘etiology’ and ‘etiologies’, or different forms of the same verb like ‘infect’, ‘infects’, ‘infected’ and ‘infecting’).
- Part-of-Speech Tagging – In this step, words are annotated with their grammatical categories, such as ‘noun’, ‘verb’, ‘adjective’ etc.
- Named-Entity Recognition – This determines which words correspond to proper names, such as person names, locations, organizations. In the interest of analyzing Medical literature, we integrate synonymy information in order to conflate alternative names of geographical entities based on the geonames^[[3]](#footnote-3)^ database, as well as alternative MESH^[[4]](#footnote-4)^ terms for the same medical concepts. For example, the word salpinx and fallopian tube(s) are conflated into the same entry.

1. Once these steps are executed, a vector space model, often called a ‘bag of words’, is built to represent relationships between all words resulting from the text preprocessing and the documents in which they occur. Simple bag of words models may rely on mere word frequencies per document. The Papyrus software leverages the TF-IDF score (short for Term Frequency-Inverse Document Frequency) which minimizes the importance of common words and gives more emphasis to discriminative words.
2. Based on the fact that the resulting bag-of-words is a sparse matrix (i.e. very few non-zero elements), Papyrus uses the CoClus^[[5]](#footnote-5)^ algorithm to extract disjoint matrix blocks corresponding to sets of paper abstracts and words which are more densely related internally than the rest of the corpus. These blocks which associate a set of documents to a tightly related set of representative words correspond in fact to extracted topics which are made of an association of semantically coherent documents and characteristic words which we call “topic-words” (e.g. an analogy in the press would be to discover automatically without prior knowledge a topic where some of the most important words are ‘covid19’, ‘lockdown’, ‘mask’, ‘PCR’, ‘vaccine’, ‘test’, ‘layoff’, ‘stimulus’, ‘bill’). Given K a target number of partitions, this algorithm tries to partition the word-document set into K parts in a way that maximizes a graph modularity metric using a heuristic with numerous iterations.
3. The extracted topics, and related documents and topic words, can then be explored using interactive visualizations.

### Supplementary text 4.2 Interactive visualization


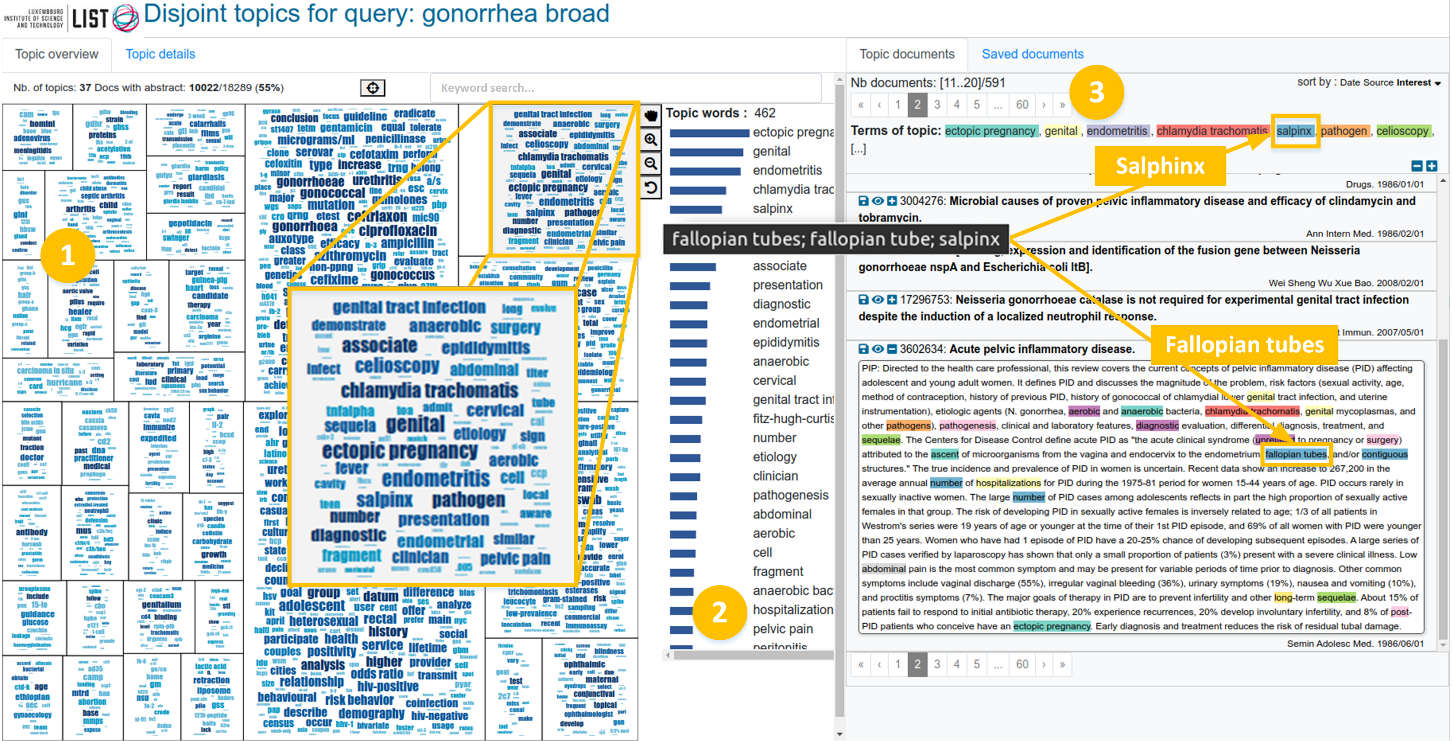


Supplementary figure 3. The main graphical user interface of Papyrus shows (1) a topic map composed of a mosaic of word clouds, (2) a bar chart showing a list of topic words ranked by importance, (3) a close reading panel where the analyst can navigate and read all relevant abstracts.

Supplementary figure 3 shows the main visual interface of Papyrus. On the left, a map of 37 topics provides a visual summary of a corpus containing 10,022 publication abstracts. When the analyst clicks on a topic, a list of topic words is displayed in the middle panel, ranked by word importance. In the right panel, a ranked list of papers supports close reading in order to better contextualize topic words. In this example, we see how alternative MESH terms salpinx and fallopian tube(s) have been merged into a single concept.

Some of the perks of Papyrus include the fact that the layout of the visual map places closely related topics in close spatial positions, based on a Multi-Dimensional Scaling (MDS)^[[6]](#footnote-6)^ projection of the topics in the 2D plane. The tool also supports keyword search to help locate the topic containing a word of interest. This is further facilitated by keyword suggestions as the analysts types the first few characters of a keyword. Additional visualizations support the analysis of word co-occurrences, which were not leveraged in the context of the present study.

### Supplementary text 4.3 Reproducibility and General Applicability of this Approach

In computer science, an algorithm is said to be deterministic if given the same input it produces the same output. In the aforementioned text processing pipeline, steps 1 and 2 are deterministic. For a given set of documents and a fixed algorithm parametrization, the system always builds the same term-document matrix. Only in step 3 ‘topic extraction’, the Coclus algorithm introduces some randomness due to the search for the most optimal value of the graph modularity metric used to assign words and documents into K topics (or matrix blocks). In the present study, this variability was circumvented by an exhaustive manual screening by two analysts of all topic words across the entire corpus. In the future, we would like to carry out a systematic study of topic stability across multiple runs of the topic extraction procedure with the same input parameters, and assess the difference between the most optimal partition and alternative partitions for a target value of K.

At the time of writing we have put in place an instance of the Papyrus software to help analyze the literature concerning the COVID19 disease, which can be accessed online at <https://colibri.list.lu/>. We are currently using Papyrus also in the process of writing a systematic literature review in computer science on high-resolution wall-sized displays. We aim to assess the validity of the principled approach described in this work and to apply it to any systematic literature review work.

1. Médoc N., Ghoniem M., Nadif M. Visual Exploration of Topic Variants Through a Hybrid Biclustering Approach. IHM '16: Actes de la 28ième conference francophone sur l'Interaction Homme-Machine. 2016; 103-114. *doi:10.1145/3004107.3004116* [↑](#footnote-ref-1)
2. *Manning C.D., Surdeanu M., Bauer J., Finkel J., Bethard S.J. and McClosky D. The Stanford CoreNLP Natural Language Processing Toolkit. ACL System Demonstrations. 2014; 55-60. doi: 10.3115/v1/P14-5010* [↑](#footnote-ref-2)
3. <https://www.geonames.org/> [↑](#footnote-ref-3)
4. <https://www.nlm.nih.gov/mesh/meshhome.html> [↑](#footnote-ref-4)
5. <https://pypi.org/project/coclust/> [↑](#footnote-ref-5)
6. Hout MC, Papesh MH, Goldinger SD.Hout MC, et al. Multidimensional scaling. Interdiscip Rev Cogn Sci. 2013 Jan;4(1):93-103. doi: 10.1002/wcs.1203. Epub 2012 Oct 8. [↑](#footnote-ref-6)
