# Supplementary material for: Applying a novel approach to scoping review incorporating artificial intelligence: mapping the natural history of gonorrhoea
Source: BMC Med Res Methodol. 2021 Sep 6;21:183. doi: 10.1186/s12874-021-01367-x (PMC8418964; doi:10.1186/s12874-021-01367-x)
Supplement: Supplementary file 5 — Additional file 5: Supplementary Text 5.1. Full list of health outcomes identified through the combination of the AI and other searches, with references and SIGN scores. Supplementary Text 5.2. List of extracted ‘topic words’ related references and health outcomes identified through AI-assisted literature search. [file 12874_2021_1367_MOESM5_ESM.docx]

## Additional file 5

### Supplementary text 5.1 Full list of health outcomes identified through combined AI and other searches, with references and SIGN score

| **Sex** | **Primary anatomic site infected** | **Final list, complications and health outcomes related to gonorrhea** | **Author** | **Journal** | **Year of publication** | **PMID** | **Study type** | **SIGN Score** |
| --- | --- | --- | --- | --- | --- | --- | --- | --- |
| **Men and / or Women** | Systemic | Disseminated Gonococcal Infection | Ludivico et al. | Arthritis Rheum | 1979 | 153144 | Case series (n=17) | 3 |
|  | Systemic | Disseminated Gonococcal Infection | Rice et al. | Infect Dis Clin North Am | 2005 | 16297736 | Review | N/A |
|  | Systemic | Disseminated Gonococcal Infection | Edwards et al. | Clin Microbiol Rev | 2004 | 15489357 | Review | N/A |
|  | Systemic | Disseminated Gonococcal Infection | Edwards et al. | Crit Rev Microbiol | 2016 | 26805040 | Review | N/A |
|  | Systemic | Disseminated Gonococcal Infection | Quillin et al. | Nat Rev Microbiol | 2018 | 29430011 | Review | N/A |
|  | Systemic | Disseminated Gonococcal Infection | Almonacid-Mendoza et al. | Methods Mol Biol | 2019 | 31119630 | Review | N/A |
|  | Systemic | Fever | McCord et al. | Arch Intern Med | 1977 | 141916 | Retrospective comparative study (n=39) | 2+ |
|  | Systemic | Leukocytosis | Gelfand et al. | J Rheumatol | 1975 | 1185739 | Case series (n=84) | 3 |
|  | Systemic | Monoarthritis | Gelfand et al. | J Rheumatol | 1975 | 1185739 | Case series (n=84) | 3 |
|  | Systemic | Monoarthritis | García-De La Torre et al. | Rheum Dis Clin North Am | 2009 | 19480997 | Review | N/A |
|  | Systemic | Monoarthritis | Rouanes et al. | Rev Med Interne | 2018 | 28844395 | Case report (n=1) | 3 |
|  | Systemic | Erythematous Macules | Gelfand et al. | J Rheumatol | 1975 | 1185739 | Case series (n=84) | 3 |
|  | Systemic | Nonpainful Macules And Papules | García-De La Torre et al. | Rheum Dis Clin North Am | 2009 | 19480997 | Review | N/A |
|  | Systemic | Gonococcal Arthritis | García-De La Torre et al. | Rheum Dis Clin North Am | 2009 | 19480997 | Review | N/A |
|  | Systemic | Migratory Arthralgias | McCord et al. | Arch Intern Med | 1977 | 141916 | Retrospective comparative study (n=39) | 2+ |
|  | Systemic | Osteomyelitis | Almonacid-Mendoza et al. | Methods Mol Biol | 2019 | 31119630 | Review | N/A |
|  | Systemic | Polyarthritis | Gelfand et al. | J Rheumatol | 1975 | 1185739 | Case series (n=84) | 3 |
|  | Systemic | Polyarthritis | Rajapakse et al. | Clin Rheumatol | 1984 | 6467867 | Case report (n=1) | 3 |
|  | Systemic | Popliteal Cyst | Weiner et al. | Sex Transm Dis | 1983 | 6648748 | Case report (n=1) | 3 |
|  | Systemic | Pustules | Gelfand et al. | J Rheumatol | 1975 | 1185739 | Case series (n=84) | 3 |
|  | Systemic | Acute Gonococcal Arthritis | McCord et al. | Arch Intern Med | 1977 | 141916 | Retrospective comparative study (n=39) | 2+ |
|  | Systemic | Septic Arthritis | Terho et al. | Sex Transm Dis | 1977 | 412257 | Case report (n=1) | 3 |
|  | Systemic | Septic Arthritis | Gelfand et al. | J Rheumatol | 1975 | 1185739 | Case series (n=84) | 3 |
|  | Systemic | Septic Arthritis | García-De La Torre et al. | Rheum Dis Clin North Am | 2009 | 19480997 | Review | N/A |
|  | Systemic | Synovial Cyst Rupture | Terho et al. | Sex Transm Dis. | 1977 | 412257 | Case report (n=1) | 3 |
|  | Systemic | Vesicles | Gelfand et al. | J Rheumatol | 1975 | 1185739 | Case series (n=84) | 3 |
|  | Systemic | Acute Pericarditis | Wilson et al. | Br Heart J | 1990 | 2317408 | Case report (n=1) | 3 |
|  | Systemic | Gonococcal-Arthritis Related Mortality | García-De La Torre et al. | Rheum Dis Clin North Am | 2009 | 19480997 | Review | N/A |
|  | Systemic | Permanent Joint Damage | García-De La Torre et al. | Rheum Dis Clin North Am | 2009 | 19480997 | Review | N/A |
|  | Systemic | Back Pain | McCord et al. | Arch Intern Med | 1977 | 141916 | Retrospective comparative study (n=39) | 2+ |
|  | Systemic | Arthritis | O'Brien et al. | Medicine (Baltimore) | 1983 | 6415361 | Case series (n=49) | 3 |
|  | Systemic | Arthritis-Dermatitis Syndrome | Ludivico et al. | Arthritis Rheum | 1979 | 153144 | Case series (n=17) | 3 |
|  | Systemic | Arthritis-Dermatitis Syndrome | Edwards et al. | Clin Microbiol Rev | 2004 | 15489357 | Review | N/A |
|  | Systemic | Rash | Gelfand et al. | J Rheumatol | 1975 | 1185739 | Case series (n=84) | 3 |
|  | Systemic | Skin Lesions | Ludivico et al. | Arthritis Rheum | 1979 | 153144 | Case series (n=17) | 3 |
|  | Systemic | Skin Lesions | O'Brien et al. | Medicine (Baltimore) | 1983 | 6415361 | Case series (n=49) | 3 |
|  | Systemic | Supparative Arthritis | O'Brien et al. | Medicine (Baltimore) | 1983 | 6415361 | Case series (n=49) | 3 |
|  | Systemic | Synovitis | ICD9/10 | N/A | N/A | N/A | Not applicable | N/A |
|  | Systemic | Tenosynovitis | Gelfand et al. | J Rheumatol | 1975 | 1185739 | Case series (n=84) | 3 |
|  | Systemic | Tenosynovitis | O'Brien et al. | Medicine (Baltimore) | 1983 | 6415361 | Case series (n=49) | 3 |
|  | Systemic | Tenosynovitis | García-De La Torre et al. | Rheum Dis Clin North Am | 2009 | 19480997 | Review | N/A |
|  | Systemic | Abscess | ICD9/10 | N/A | N/A | N/A | Not applicable | N/A |
|  | Systemic | Endocarditis (+ Death) | Jackman et al. | Am J Med Sci | 1991 | 2000895 | Case report (n=5, 6 episodes) | 3 |
|  | Systemic | Endocarditis (+ Death) | Fernandez et al. | Am Heart J | 1984 | 6437201 | Case series (n=4+25 historical cases) | 3 |
|  | Systemic | Pericardial Effusion | Wilson et al. | Br Heart J. | 1990 | 2317408 | Case report (n=1) | 3 |
|  | Systemic | Pericarditis + Tamponade | Coe et al. | Arthritis Rheum. | 1990 | 2403406 | Case report (n=1) | 3 |
|  | Systemic | Meningitis (Primary Infection) | Billings et al. | STD | 1991 | 1907402 | Case report (n=1) | 3 |
|  | Systemic | Adult Respiratory Distress Syndrome | Belding et al. | Rev Infect Dis. | 1991 | 1775843 | Case report (n=1) | 3 |
|  | Systemic | Adult Respiratory Distress Syndrome | Almonacid-Mendoza et al. | Methods Mol Biol | 2019 | 31119630 | Review | N/A |
|  | Systemic | Brain Abscess | ICD9/10 | N/A | N/A | N/A | Not applicable | N/A |
|  | Systemic | Bursitis | ICD9/10 | N/A | N/A | N/A | Not applicable | N/A |
|  | Systemic | Cellulitis | ICD9/10 | N/A | N/A | N/A | Not applicable | N/A |
|  | Systemic | Endocarditis | Edwards et al. | Clin Microbiol Rev | 2004 | 15489357 | Review | N/A |
|  | Systemic | Endocarditis | Belkacem et al. | Sex Transm Infect. | 2013 | 23920397 | Case series (n=21) | 3 |
|  | Systemic | Endocarditis | Quillin et al. | Nat Rev Microbiol | 2018 | 29430011 | Review | N/A |
|  | Systemic | Epidural Abscess | van Hal et al. | Med J Aust. | 2004 | 14709128 | Case report (n=1) | 3 |
|  | Systemic | Paravertebral Abscess | Low et al. | J Neurosurg Spine | 2012 | 22578237 | Case report (n=1) | 3 |
|  | Systemic | Glomerularnephritis | Noor et al. | BMJ Case Rep. | 2018 | 30021740 | Case report (n=1) | 3 |
|  | Systemic | Heart Infection | ICD9/10 | N/A | N/A | N/A | Not applicable | N/A |
|  | Systemic | Hepatitis | ICD9/10 | N/A | N/A | N/A | Not applicable | N/A |
|  | Systemic | Infectious Arthritis | Quillin et al. | Nat Rev Microbiol | 2018 | 29430011 | Review | N/A |
|  | Systemic | Keratosis | ICD9/10 | N/A | N/A | N/A | Not applicable | N/A |
|  | Systemic | Liver Capsule (Fitzhugh-Curtis Syndrome) | Ndowa et al. | Reprod Health Matters. | 2012 | 23245412 | Review | N/A |
|  | Systemic | Meningitis | Edwards et al. | Clin Microbiol Rev | 2004 | 15489357 | Review | N/A |
|  | Systemic | Meningitis | Almonacid-Mendoza et al. | Methods Mol Biol | 2019 | 31119630 | Review | N/A |
|  | Systemic | Mono / Polyarthralgia | O'Brien et al. | Medicine (Baltimore) | 1983 | 6415361 | Case series (n=49) | 3 |
|  | Systemic | Ovarian Capsule Hemorrhage | Schneider et al. | Geburtshilfe Frauenheilkd. | 1995 | 7607387 | Case report (n=1) | 3 |
|  | Systemic | Pericardial Tamponade | Coe et al. | Arthritis Rheum. | 1990 | 2403406 | Case report (n=1) | 3 |
|  | Systemic | Pericarditis | Coe et al. | Arthritis Rheum. | 1990 | 2403406 | Case report (n=1) | 3 |
|  | Systemic | Perihepatitis | ICD9/10 | N/A | N/A | N/A | Not applicable | N/A |
|  | Systemic | Peritoneal Hemorrhage | Schneider et al. | Geburtshilfe Frauenheilkd. | 1995 | 7607387 | Case report (n=1) | 3 |
|  | Systemic | Peritonitis | ICD9/10 | N/A | N/A | N/A | Not applicable | N/A |
|  | Systemic | Rhabdomyolysis (Rml) | Attorri et al. | Medicina (B Aires). | 1998 | 10347969 | Case report (n=1) | 3 |
|  | Systemic | Septic Shock | Almonacid-Mendoza et al. | Methods Mol Biol | 2019 | 31119630 | Review | N/A |
|  | Systemic | Septicaemia/Sepsis | ICD9/10 | N/A | N/A | N/A | Not applicable | N/A |
|  | Systemic | Septicaemia/Sepsis | Read Code | N/A | N/A | N/A | Not applicable | N/A |
|  | Systemic | Spondylitis | ICD9/10 | N/A | N/A | N/A | Not applicable | N/A |
|  | Systemic | Subcutaneous Abscess | Owino et al. | Br J Vener Dis. | 1981 | 7214122 | Case report (n=1) | 3 |
|  | Systemic | Vasculitis | Jain et al. | J Clin Path | 2007 | 17213353 | Case report (n=1) | 3 |
|  | Systemic | Aortic Aneurysm | Risher et al. | Ann Thorac Surg | 1994 | 8147654 | Case report (n=1) | 3 |
|  | Systemic | Aortitis | Woo et al. | Ann Thorac Surg | 2011 | 21353028 | Case report (n=1) | 3 |
|  | Systemic | Antibiotic Resistance + Increased Incidence | Chesson et al. | Emerg Infect Dis | 2004 | 24655615 | Trend analysis | 2- |
|  | Oropharygeal | Mucosal Infection Pharynx | Edwards et al. | Crit Rev Microbiol | 2016 | 26805040 | Review | N/A |
|  | Oropharygeal | Asymptomatic Pharyngeal Infection | Ndowa et al. | Reprod Health Matters. | 2012 | 23245412 | Review | N/A |
|  | Oropharygeal | Pharyngitis | Almonacid-Mendoza et al. | Methods Mol Biol | 2019 | 31119630 | Review | N/A |
|  | Oropharygeal | Gingivitis | Holmstrup et al. | Ann Periodontol | 1999 | 10863372 | Review | N/A |
|  | Oropharygeal | Gingivitis | Almonacid-Mendoza et al. | Methods Mol Biol | 2019 | 31119630 | Review | N/A |
|  | Oropharygeal | Intraoral And Cutaneous Abscess Formation | Almonacid-Mendoza et al. | Methods Mol Biol | 2019 | 31119630 | Review | N/A |
|  | Eye | Anterior Chamber Inflammation | Wan et al. | Am J Ophthalmol | 1986 | 3777076 | Case series (n=21) | 3 |
|  | Eye | Conjunctivitis | Edwards et al. | Crit Rev Microbiol | 2016 | 26805040 | Review | N/A |
|  | Eye | Conjunctivitis | Almonacid-Mendoza et al. | Methods Mol Biol | 2019 | 31119630 | Review | N/A |
|  | Eye | Iridocylitis | ICD9/10 | N/A | N/A | N/A | N/A | N/A |
|  | Eye | Irritation / Pain | Wan et al. | Am J Ophthalmol | 1986 | 3777076 | Case series (n=21) | 3 |
|  | Eye | Keratitis | Wan et al. | Am J Ophthalmol | 1986 | 3777076 | Case series (n=21) | 3 |
|  | Eye | Keratoconjunctivitis | Schwab et al. | Br J Ophthalmol. | 1985 | 4016048 | Outbreak investigation (n=16) | 3 |
|  | Eye | Periocular Edema And Tenderness, | Wan et al. | Am J Ophthalmol | 1986 | 3777076 | Case series (n=21) | 3 |
|  | Eye | Preauricular Lymphadenopathy | Wan et al. | Am J Ophthalmol | 1986 | 3777076 | Case series (n=21) | 3 |
|  | Eye | Purulent Discharge | Wan et al. | Am J Ophthalmol | 1986 | 3777076 | Case series (n=21) | 3 |
|  | Eye | Neonatal Blindness | Ndowa et al. | Reprod Health Matters. | 2012 | 23245412 | Review | N/A |
|  | Eye | Visual Acuity Of Light Perception | Wan et al. | Am J Ophthalmol | 1986 | 3777076 | Case series (n=21) | 3 |
|  | Eye | Corneal Perforation | Kawashima et al. | Eye (Lond). | 2009 | 18064057 | Case report (n=5) | 3 |
|  | Eye | Endopthalmitis | Quirke et al. | International Journal of Infectious Diseases | 2008 | 18093859 | Case series (n=51) | 3 |
|  | Eye | Gaze Restriction | Wan et al. | Am J Ophthalmol | 1986 | 3777076 | Case series (n=21) | 3 |
|  | Co-infection | HIV Infection | Ndowa et al. | Reprod Health Matters. | 2012 | 23245412 | Review | N/A |
|  | Co-infection | Increased HIV-1 Viral Shedding | Marrazzo et al. | Infect Dis Clin North Am | 2005 | 15963875 | Review | N/A |
|  | Co-infection | *M. Genitalium / U. Urealyticum.* | Yokoi et al. | CID | 2007 | 17806051 | Cross-sectional study of association (n=390) | 2- |
|  | Co-infection | Susceptibility To HIV | Edwards et al. | Clin Microbiol Rev | 2004 | 15489357 | Review | N/A |
|  | Anorectal | Asymptomatic Proctitis | Ghanem et al. | Up to date | 2018 | https://www.uptodate.com/contents/clinical-manifestations-and-diagnosis-of-neisseria-gonorrhoeae-infection-in-adults-and-adolescents | Review | N/A |
|  | Anorectal | Mucosal Infection Rectum | Edwards et al. | Crit Rev Microbiol | 2016 | 26805040 | Review | N/A |
|  | Anorectal | Proctitis | Ghanem et al. | Up to date | 2018 | https://www.uptodate.com/contents/clinical-manifestations-and-diagnosis-of-neisseria-gonorrhoeae-infection-in-adults-and-adolescents | Review | N/A |
|  | Anorectal | Anal Fissures | Travassos et al. | Braz J Infect Dis. | 2016 | 27765581 | Cross-sectional study of association (n=521) | 2- |
|  | Anorectal | Anovaginal Fistula | Barre et al. | Presse Med | 2018 | 29909937 | Case report (n=1) | 3 |
|  | Anorectal | Perianal Abscess | El-Dhuwaib et al. | Eur J Clin Microbiol Infect Dis. | 2003 | 12827535 | Case report (n=1) | 3 |
|  | Psychosocial | Fear Of Loss Of Relationship | Cunningham et al. | Sex Health | 2007 | 17382038 | Qualitative study (n=21 female adolescents) | 3 |
|  | Psychosocial | Stigmatisation | Cunningham et al. | Sex Health | 2007 | 17382038 | Qualitative study (n=21 female adolescents) | 3 |
|  | Psychosocial | Lack Of Knowledge About Gonorrhoea | Biro et al. | Clin Pediatr | 1994 | 7813139 | Cross-sectional survey (n=248) | 2- |
|  | Urogenital | Pelveoperitonitis | Gurlich et al. | Prague Med Rep | 2005 | 16572933 | Case report (n=1) | 3 |
| **Men** | Urogenital | Epididymitis, Acute | Almonacid-Mendoza et al. | Methods Mol Biol | 2019 | 31119630 | Review | N/A |
|  | Urogenital | Azoospermia | Ndowa et al. | Reprod Health Matters. | 2012 | 23245412 | Review | N/A |
|  | Urogenital | Infertility (Men) | Han et al. | Andrologia. | 2016 | 26688510 | Cross-sectional study of association in OA patients (n=133) | 2- |
|  | Urogenital | Obstructive Azoospermia (Oa) | Han et al. | Andrologia. | 2016 | 26688510 | Cross-sectional study of association in OA patients (n=133) | 2- |
|  | Urogenital | Deferentitis | Kornelishin et al. | Vestn Dermatol Venerol. | 1990 | 2256383 | Case series (n=28) | 3 |
|  | Urogenital | Epididymo-Orchitis | ICD9/10 | N/A | N/A | N/A | Not applicable | N/A |
|  | Urogenital | Epididymo-Orchitis | Kornelishin et al. | Vestn Dermatol Venerol. | 1990 | 2256383 | Case series (n=28) | 3 |
|  | Urogenital | Epididymo-Orchitis | Mensforth et al. | Int J STD AIDS. | 2018 | 28705094 | Prevalence study (EO, n=63) | 3 |
|  | Urogenital | Epidydimitis, Chronic | Kornelishin et al. | Vestn Dermatol Venerol. | 1990 | 2256383 | Case series (n=28) | 3 |
|  | Urogenital | Funiculitis | Kornelishin et al. | Vestn Dermatol Venerol. | 1990 | 2256383 | Case series (n=28) | 3 |
|  | Urogenital | Orchitis | ICD9/10 | N/A | N/A | N/A | Not applicable | N/A |
|  | Urogenital | Penile Lymphangitis | Almonacid-Mendoza et al. | Methods Mol Biol | 2019 | 31119630 | Review | N/A |
|  | Urogenital | Periurethral Abscess | Almonacid-Mendoza et al. | Methods Mol Biol | 2019 | 31119630 | Review | N/A |
|  | Urogenital | Scrotal / Perineal Abscesses | Kleist et al. | Ugeskr Laeger | 1993 | 1462439 | Case series (n=46) | 3 |
|  | Urogenital | Seminal Vesiculitis | Almonacid-Mendoza et al. | Methods Mol Biol | 2019 | 31119630 | Review | N/A |
|  | Urogenital | Testicular Abscess +.- Death Of Muscle and Tissue Of Testis | Ndowa et al. | Reprod Health Matters. | 2012 | 23245412 | Review | N/A |
|  | Urogenital | Acute Prostatitis | Kornelishin et al. | Vestn Dermatol Venerol. | 1990 | 2256383 | Case series (n=28) | 3 |
|  | Urogenital | Acute Prostatitis | Belkacem et al. | Sex Transm Infect. | 2013 | 23920397 | Case series (n=21) | 3 |
|  | Urogenital | Acute Prostatitis | Almonacid-Mendoza et al. | Methods Mol Biol | 2019 | 31119630 | Review | N/A |
|  | Urogenital | Infection Of The Prostate | De Marzo et al. | Prostate | 2017 | 28703328 | Case control study (n=1091) | 2+ |
|  | Urogenital | Prostatovesiculitis | Kornelishin et al. | Vestn Dermatol Venerol. | 1990 | 2256383 | Case series (n=28) | 3 |
|  | Urogenital | Prostate Cancer | Heshmat et al. | Urology | 1975 | 1179565 | Case control study (n=150) | 2- |
|  | Urogenital | Prostate Cancer | Sutcliffe et al. | Cancer Epidemiol Biomarkers Prev | 2006 | 17119041 | Prospective cohort study (n=36033) | 2+ |
|  | Urogenital | Prostate Cancer | Wang et al. | Eur J Clin Microbiol Infect dis | 2017 | 28013414 | Retrospective cohort study (n=1775) | 2+ |
|  | Urogenital | Prostatitis | Sutcliffe et al. | Am J Epidemiol | 2005 | 16177142 | Prospective cohort study (n=32932) | 2+ |
|  | Urogenital | Acute Urethritis | Edwards et al. | Cell Microbiol | 2004 | 11553013 | Review | N/A |
|  | Urogenital | Asymptomatic Urethral Infection | Ndowa et al. | Reprod Health Matters. | 2012 | 23245412 | Review | N/A |
|  | Urogenital | Dysuria | Shapiro et al. | Acad Emerg Med | 2005 | 15635136 | Prospective cohort study (n=92) | 2- |
|  | Urogenital | Dysuria | Almonacid-Mendoza et al. | Methods Mol Biol | 2019 | 31119630 | Review | N/A |
|  | Urogenital | Haematuria | Amarasuriya et al. | Br J Vener Dis | 1979 | 380776 | Case series (n=50) | 3 |
|  | Urogenital | Nocturia | Sutcliffe et al. | Am J Epidemiol | 2005 | 16177142 | Prospective cohort study (n=32932) | 2+ |
|  | Urogenital | Urethral Discharge | Matteelli et al. | STI | 2000 | 10858716 | Syndromic surveillance report | 3 |
|  | Urogenital | Urethral Discharge | Almonacid-Mendoza et al. | Methods Mol Biol | 2019 | 31119630 | Review | N/A |
|  | Urogenital | Urethritis In Men | Ludivico et al. | Arthritis Rheum | 1979 | 153144 | Case series (n=17) | 3 |
|  | Urogenital | Urethritis In Men | Terho et al. | Sex Transm Dis. | 1977 | 412257 | Case report (n=1) | 3 |
|  | Urogenital | Urethritis In Men | Shaughnessy et al. | Methods Mol Biol | 2019 | 31119614 | Review | N/A |
|  | Urogenital | Chronic Urethritis | Read Code | N/A | N/A | N/A | Not applicable | N/A |
|  | Urogenital | Reduced Renal Function | Kleist et al. | Ugeskr Laeger | 1992 | 1462439 | Case series (n=46) | 3 |
|  | Urogenital | Urethral Fistulae | Osoba et al. | Br J Vener Dis | 1976 | 1009418 | Case series (n=16) | 3 |
|  | Urogenital | Urethral Stricture | Kleist et al. | Ugeskr Laeger | 1994 | 1462439 | Case series (n=46) | 3 |
|  | Co-infection | HIV In Men | Beck et al. | Int J STD AIDS | 1996 | 8652708 | Case control study (n=1168 men) | 2- |
|  | Co-infection | HIV In MSM | Beymer et al. | J Acquir Immune Defic Syndr | 2016 | 27163174 | Prospective cohort study (n=3111) | 2- |
|  | Co-infection | HIV In MSM | Barbee et al. | STD | 2017 | 28608786 | Case control study (n=880) | 2- |
| **Women** | Urogenital | Invasive Vulval Carcinoma | Sherman et al. | Epidemiology | 1991 | 1655066 | Case-control study (n=646) | 2- |
|  | Urogenital | Vulval Carcinoma In Situ | Sherman et al. | Epidemiology | 1991 | 1655066 | Case-control study (n=646) | 2- |
|  | Urogenital | Cervical Carcinoma | Sandmire et al. | Obstet Gynecol | 1976 | 934574 | Laboratory surveillance study (n=40,000) | 3 |
|  | Urogenital | Cervical Intraepithelial Neoplasia Grade Iii | Johansen et al. | Acta Obstet Gynecol Scand | 2001 | 11531621 | Registry data (n=4440) | 2- |
|  | Urogenital | Invasive Cervical Cancer | de Sanjose et al. | Int J Cancer. | 1994 | 8314322 | Case-control study (n=1037) | 2+ |
|  | Urogenital | Chronic Endometritis | Laniewski et al. | Methods Mol Biol | 2019 | 31119633 | Review | 2+ |
|  | Urogenital | Chronic Pelvic Pain | Shaughnessy et al. | Methods Mol Biol | 2019 | 31119614 | Review | N/A |
|  | Urogenital | Ectopic Pregnancy | Kallenberger et al. | South Med J. | 1978 | 663716 | Case series (n=160) | 3 |
|  | Urogenital | Ectopic Pregnancy | Ankum et al. | Fertil Steril. | 1996 | 8641479 | Meta-analysis (4 case-control studies) | 1- |
|  | Urogenital | Ectopic Pregnancy | Edwards et al. | Cell Microbiol | 2004 | 11553013 | Review | N/A |
|  | Urogenital | Ectopic Pregnancy | Moore et al. | STD | 2016 | 26656441 | Trend analysis | 2- |
|  | Urogenital | Ectopic Pregnancy | Quillin et al. | Nat Rev Microbiol | 2018 | 29430011 | Review | N/A |
|  | Urogenital | Ectopic Pregnancy | Shaughnessy et al. | Methods Mol Biol | 2019 | 31119614 | Review | N/A |
|  | Urogenital | Infertility (Women) | Edwards et al. | Cell Microbiol | 2004 | 11553013 | Review | N/A |
|  | Urogenital | Infertility (Women) | Edwards et al. | Clin Microbiol Rev | 2004 | 15489357 | Review | N/A |
|  | Urogenital | Infertility (Women) | Morales et al. | Infect Immun | 2006 | 16714596 | Review | N/A |
|  | Urogenital | Infertility (Women) | Tsevat et al. | Am J Obstet Gynecol | 2017 | 28007229 | Review | N/A |
|  | Urogenital | Infertility (Women) | Quillin et al. | Nat Rev Microbiol | 2018 | 29430011 | Review | N/A |
|  | Urogenital | Intra-abdominal Adhesions & Chronic Pelvic Pain | Lenz et al. | Front Immunol | 2018 | 30524442 | Review | N/A |
|  | Urogenital | Occlusion Of The Oviduct | Lenz et al. | Front Immunol | 2018 | 30524442 | Review | N/A |
|  | Urogenital | Tubal Factor Infertility | Lenz et al. | Front Immunol | 2018 | 30524442 | Review | N/A |
|  | Urogenital | Tubal Factor Infertility | Shaughnessy et al. | Methods Mol Biol | 2019 | 31119614 | Review | N/A |
|  | Urogenital | Tubal Infertililty | Grodstein et al. | Am J Epidemiology | 1993 | 8465809 | Case-control study (n=4116) | 2- |
|  | Urogenital | Tubal Infertility | Sherman et al. | STD | 1987 | 3563829 | Case control study (n=979) | 2- |
|  | Urogenital | Tubal Scarring | Edwards et al. | Clin Microbiol Rev | 2004 | 15489357 | Review | N/A |
|  | Urogenital | Tubal Scarring | Lenz et al. | Front Immunol | 2018 | 30524442 | Review | N/A |
|  | Urogenital | Tubo-Ovarian Abscess | Miller et al. | Am Fam Physician | 2006 | 16734055 | Review | N/A |
|  | Urogenital | Tubal Infertililty | Cates et al. | Am J Obstet Gynecol | 1993 | 8362945 | Case-control study (n=1912) | 2- |
|  | Urogenital | Acute Perihepatitis (Fitz-Hugh–Curtis Syndrome) | Almonacid-Mendoza et al. | Methods Mol Biol | 2019 | 31119630 | Review | N/A |
|  | Urogenital | Cervicitis (Mucopurulent) | Morales et al. | Infect Immun | 2006 | 16714596 | Review | N/A |
|  | Urogenital | Co-Infection (Chlamydia) | Seigel et al. | J Adolesc Health. | 1995 | 7742335 | Laboratory surveillance study (n=1990) | 2- |
|  | Urogenital | Disseminated Gonococcal Infection in Pregnancy | Phupong et al. | Arch Gynecol Obstet | 2005 | 16136359 | Case report (n=1) | 3 |
|  | Urogenital | Ectopic Pregnancy | Edwards et al. | Clin Microbiol Rev | 2004 | 15489357 | Review | N/A |
|  | Urogenital | Ectopic Pregnancy | Lenz et al. | Front Immunol | 2018 | 30524442 | Review | N/A |
|  | Urogenital | Endocarditis | Almonacid-Mendoza et al. | Methods Mol Biol | 2019 | 31119630 | Review | N/A |
|  | Urogenital | Endocervical Mucopus | Chandeying et al. | STI | 1998 | 9849555 | Prospective study: clinical protocol evaluation, non-random allocation (n=240) | 2- |
|  | Urogenital | Endocervicitis | Kornelishin et al. | Vestn Dermatol Venerol. | 1990 | 2256383 | Case series (n=28) | 3 |
|  | Urogenital | Endometritis | Kornelishin et al. | Vestn Dermatol Venerol. | 1990 | 2256383 | Case series (n=28) | 3 |
|  | Urogenital | Endometritis | Timmerman et al. | Cell Microbiol | 2005 | 15839892 | Review | N/A |
|  | Urogenital | Endometritis | Morales et al. | Infect Immun | 2006 | 16714596 | Review | N/A |
|  | Urogenital | Endometritis | Shaughnessy et al. | Methods Mol Biol | 2019 | 31119614 | Review | N/A |
|  | Urogenital | Endometritis | Almonacid-Mendoza et al. | Methods Mol Biol | 2019 | 31119630 | Review | N/A |
|  | Urogenital | Endometritis + Salpingitis | Plummer et al. | J Infect Dis | 1987 | 3598229 | Prospective cohort study (n=1,013) | 2- |
|  | Urogenital | Endomyometritis | Kornelishin et al. | Vestn Dermatol Venerol. | 1990 | 2256383 | Case series (n=28) | 3 |
|  | Urogenital | Fallopian Tubal Abscess | Morales et al. | Infect Immun | 2006 | 16714596 | Review | N/A |
|  | Urogenital | Fallopian Tube Salpingitis | Almonacid-Mendoza et al. | Methods Mol Biol | 2019 | 31119630 | Review | N/A |
|  | Urogenital | Gonococcal Urethritis | Edwards et al. | Clin Microbiol Rev | 2004 | 15489357 | Review | N/A |
|  | Urogenital | Pelvic (Tubal Or Ovarian) Peritonitis | Morales et al. | Infect Immun | 2006 | 16714596 | Review | N/A |
|  | Urogenital | Pelvic Inflammatory Disease | Laniewski et al. | Methods Mol Biol | 2019 | 31119633 | Review | N/A |
|  | Urogenital | Pelvic Inflammatory Disease | Edwards et al. | Clin Microbiol Rev | 2004 | 15489357 | Review | N/A |
|  | Urogenital | Pelvic Inflammatory Disease | Kamwendo et al. | Sex Transm Dis. | 1996 | 8885069 | Trend analysis | 2- |
|  | Urogenital | Pelvic Inflammatory Disease | Edwards et al. | Cell Microbiol | 2004 | 11553013 | Review | N/A |
|  | Urogenital | Pelvic Inflammatory Disease | Morales et al. | Infect Immun | 2006 | 16714596 | Review | N/A |
|  | Urogenital | Pelvic Inflammatory Disease | Moore et al. | STD | 2016 | 26656441 | Trend analysis | 2- |
|  | Urogenital | Pelvic Inflammatory Disease | Reekie et al. | Clin Infect Dis | 2018 | 29136127 | Retrospective cohort study (3199135) | 2+ |
|  | Urogenital | Pelvic Inflammatory Disease | Quillin et al. | Nat Rev Microbiol | 2018 | 29430011 | Review | N/A |
|  | Urogenital | Pelvic Inflammatory Disease | Lenz et al. | Front Immunol | 2018 | 30524442 | Review | N/A |
|  | Urogenital | Pelvic Inflammatory Disease | Shaughnessy et al. | Methods Mol Biol | 2019 | 31119614 | Review | N/A |
|  | Urogenital | Pelvic Inflammatory Disease | Almonacid-Mendoza et al. | Methods Mol Biol | 2019 | 31119630 | Review | N/A |
|  | Urogenital | Salpingitis | Soper et al. | Am J Obstet Gynaecol | 1992 | 1530018 | Cross-sectional study (n=36) | 3 |
|  | Urogenital | Salpingitis | Morales et al. | Infect Immun | 2006 | 16714596 | Review | N/A |
|  | Urogenital | Salpingitis | Shaughnessy et al. | Methods Mol Biol | 2019 | 31119614 | Review | N/A |
|  | Urogenital | Salpingitis +/- Endometritis | Plummer et al. | J Infect Dis | 1987 | 3598229 | Prospective cohort study (n=1,013) | 2- |
|  | Urogenital | Severe Hemorrhage Post Ectopic Pregnancy | Shaughnessy et al. | Methods Mol Biol | 2019 | 31119614 | Review | N/A |
|  | Urogenital | Adverse Pregnancy Outcomes | Edwards et al. | Crit Rev Microbiol | 2016 | 26805040 | Review | N/A |
|  | Urogenital | Fetal Wastage | Edwards et al. | Crit Rev Microbiol | 2016 | 26805040 | Review | N/A |
|  | Urogenital | Infant Death | Warr et al. | Sex Transm Infect | 2019 | 30228109 | Nested longitudinal analysis (n=1221) | 2- |
|  | Urogenital | Low Birth Weight | Heumann et al. | Sex Transm Dis | 2017 | 28407641 | Retrospective cohort study (n=819) | 2- |
|  | Urogenital | Miscarriage | Campbell et al. | Aust N Z J Public Health | 2011 | 21806729 | Retrospective cohort study (n=961) | 2+ |
|  | Urogenital | Premature Rupture Of Membranes | Amstey et al. | J Am Vener Dis Assoc | 1976 | 1010761 | Retrospective cohort study (n=5065) | 2- |
|  | Urogenital | Premature Rupture Of Membranes | Woods et al. | Semin Pediatr Infect Dis | 2005 | 16210106 | Review | N/A |
|  | Urogenital | Preterm Delivery | Woods et al. | Semin Pediatr Infect Dis | 2005 | 16210106 | Review | N/A |
|  | Urogenital | Preterm Delivery | Liu et al. | Sex Transm Infect | 2013 | 24005255 | Retrospective cohort study (n=354217) | 2+ |
|  | Urogenital | Preterm Delivery | Waight et al. | J La State Med Soc | 2013 | 24133786 | Database study (surveillance data) | 2+ |
|  | Urogenital | Preterm Delivery | Shaughnessy et al. | Methods Mol Biol | 2019 | 31119614 | Review | N/A |
|  | Urogenital | Preterm Delivery | Mann et al. | J Maternal-fetal & Neonatal Medicine | 2010 | 19903113 | Cohort study (n=108346) | 2- |
|  | Urogenital | Prolonged Rupture Of Membranes | Amstey et al. | J Am Vener Dis Assoc | 1976 | 1010761 | Retrospective cohort study (n=5065) | 2- |
|  | Urogenital | Prolonged Rupture Of Membranes | Ekwo et al. | Int J Epidemiol | 1993 | 8359967 | Case-control study (n=368) | 2- |
|  | Urogenital | Septic Abortion | Woods et al. | Semin Pediatr Infect Dis | 2005 | 16210106 | Review | N/A |
|  | Urogenital | Small For Gestational Age | Heumann et al. | Sex Transm Dis | 2017 | 28407641 | Retrospective cohort study (n=819) | 2- |
|  | Urogenital | Small For Gestational Age | Reekie et al. | Lancet Infect Dis | 2018 | 29371067 | Retrospective cohort study (3199135) | 2- |
|  | Urogenital | Stillbirth | Liu et al. | Sex Transm Infect | 2013 | 24005255 | Retrospective cohort study (n=354217) | 2+ |
|  | Urogenital | Stillbirth | Moodley et al. | Sex Transm Infect | 2017 | 28396556 | Retrospective observational study (n=615) | 2- |
|  | Urogenital | Cystitis | ICD9/10 | N/A | N/A | N/A | Not applicable | N/A |
|  | Urogenital | Urgency | Shapiro et al. | Acad Emerg Med | 2005 | 15635136 | Prospective cohort study (n=92) | 2- |
|  | Urogenital | Urinary Frequency | Shapiro et al. | Acad Emerg Med | 2005 | 15635136 | Prospective cohort study (n=92) | 2- |
|  | Urogenital | Asymptomatic Cervicitis | Suzuki et al. | PLoS One | 2019 | 30730922 | Prevalence study (n=281 institutes) | 3 |
|  | Urogenital | Cervicitis, Acute | Edwards et al. | Clin Microbiol Rev | 2004 | 15489357 | Review | N/A |
|  | Urogenital | Cervicitis, Acute | Shaughnessy et al. | Methods Mol Biol | 2019 | 31119614 | Review | N/A |
|  | Urogenital | Cervicitis, Acute | Almonacid-Mendoza et al. | Methods Mol Biol | 2019 | 31119630 | Review | N/A |
|  | Urogenital | Mucopurulent Cervicitis | Marrazzo et al. | Infect Dis Clin North Am | 2005 | 15963875 | Review | N/A |
|  | Urogenital | Vaginal Discharge | Matteelli et al. | STI | 2000 | 10858716 | Syndromic surveillance report | 3 |
|  | Urogenital | Vulvovaginitis | ICD9/10 | N/A | N/A | N/A | Not applicable | N/A |
|  | Urogenital | Cervicitis, Chronic | ICD9/10 | N/A | N/A | N/A | Not applicable | N/A |
|  | Urogenital | Bartholin'S Abscess | Bleker et al. | Genitourin Med | 1990 | 2107139 | Case series (n=5) | 3 |
|  | Urogenital | Bartholin'S Cyst | Bleker et al. | Genitourin Med | 1990 | 2107139 | Case series (n=5) | 3 |
|  | Urogenital | Bartholin'S Disorder | Bleker et al. | Genitourin Med | 1990 | 2107139 | Case series (n=5) | 3 |
|  | Urogenital | Bartholonitis/Bartholin’S Gland Abscess | Ghanem et al. | Up to date | 2018 | N/A | Review | N/A |
|  | Urogenital | Dyspareunia | Moran et al. | BMJ Clin Evid | 2007 | 19454057 | Systematic review (21 systematic review, RCTs or observational studies included) | 2++ |
|  | Co-infection | HIV In Women | Laga et al. | AIDS | 1993 | 8442924 | Nested case-control study (n=431) | 2- |
|  | Co-infection | HIV In Women | Peterman et al. | Int J STD AIDS | 2015 | 24713228 | Retrospective cohort using surveillance data | 2- |
|  | Co-infection | HIV In Women | Newman et al. | Clin Infect Dis. | 2019 | 30976788 | Retrospective cohort using registry data | 2- |
|  | Co-infection | HIV In Women | Beck et al. | Int J STD AIDS | 1996 | 8652708 | Case control study (n=105 women) | 2- |
|  | Co-infection | Bactervial Vaginosis | Gallo et al. | Ann Epidemiol | 2012 | 22192490 | Prospective cohort study (n=645) | 2- |
|  | Co-infection | Co-Infection (Chlaymdia) | Siegel et al. | J Adolesc Health | 1995 | 7742335 | Laboratory surveillance study (n=1990) | 2- |
| **Newborn** | Neonatal infection | Gonococcal Colonization Of The Oropharynx +/- Gastric Fluid | Woods et al. | Semin Pediatr Infect Dis | 2005 | 16210106 | Review | N/A |
|  | Neonatal infection | Gonococcal Ophthalmia Neonatorum | Lepage et al. | J Antimicrob Chemother | 1990 | 2228841 | Case series (n=21) | 3 |
|  | Neonatal infection | Gonococcal Ophthalmia Neonatorum | Laga et al. | Lancet | 1986 | 2877285 | Prospective cohort study (n=1,019) | 2+ |
|  | Neonatal infection | Gonococcal Ophthalmia Neonatorum | Woods et al. | Semin Pediatr Infect Dis | 2005 | 16210106 | Review | N/A |
|  | Neonatal infection | Gonococcal Ophthalmia Neonatorum | Edwards et al. | Crit Rev Microbiol | 2016 | 26805040 | Review | N/A |
|  | Neonatal infection | Gonococcal Ophthalmia Neonatorum | Almonacid-Mendoza et al. | Methods Mol Biol | 2019 | 31119630 | Review | N/A |
|  | Neonatal infection | Neonatal Inflammation Of Mucosal Membranes | Woods et al. | Semin Pediatr Infect Dis | 2005 | 16210106 | Review | N/A |
|  | Neonatal infection | Scalp Abscesses | Woods et al. | Semin Pediatr Infect Dis | 2005 | 16210106 | Review | N/A |
|  | Neonatal infection | Wound Infections | Woods et al. | Semin Pediatr Infect Dis | 2005 | 16210106 | Review | N/A |
|  | Neonatal infection | Neonatal Blindness | Quillin et al. | Nat Rev Microbiol | 2018 | 29430011 | Review | N/A |
|  | Neonatal infection | Systemic Disease (Sepsis, Meningitis) | Woods et al. | Semin Pediatr Infect Dis | 2005 | 16210106 | Review | N/A |

AI, artificial intelligence; ICD 9/10, International Statistical Classification of Diseases and Related Health Problems (Ninth or Tenth Revision); SIGN, Scottish Intercollegiate Guidelines Network; PMID, Pubmed identification number

### Supplementary text 5.2 Full list of topic words extracted from PAPYRUS (n=102), related references and health outcomes identified (n=124) through AI literature search.

| **Topic words extracted from Papyrus** | **PMID** | **Author** | **Journal** | **Year of publication** | **Disease subgroup assigned by the authors (JW and EB)** |
| --- | --- | --- | --- | --- | --- |
| pericarditis | 2317408 | Wilson | Br Heart J | 1990 | Acute Pericarditis |
| drug resistance, microbial | 24655615 | Chesson | Emerg Infect Dis | 2004 | Antimicrobial resistance |
| aortic aneurysm | 8147654 | Risher | Ann Thorac Surg | 1994 | aortic aneurysm |
| aortitis | 21353028 | Woo | Ann Thorac Surg | 2011 | aortitis |
| gonococcemia | 1775843 | Belding | Rev Infect Dis | 1991 | ARDS |
| arthritic | 141916 | McCord | Arch Intern Med | 1977 | Back pain |
| bactervial vaginosis | 22192490 | Gallo | Ann Epidemiol | 2012 | bacterial vaginosis |
| Bartholin's gland abcess | 2107139 | Bleker | Genitourin Med | 1990 | Bartholin's abscess |
| Bartholin's gland cyst | 2107139 | Bleker | Genitourin Med | 1990 | Bartholin's abscess |
| disorder | 2107139 | Bleker | Genitourin Med | 1990 | Bartholin's abscess |
| carcinoma | 934574 | Sandmire | Obstet Gynecol | 1976 | cervical carcinoma |
| Cervical intraepithelial neoplasia grade 3 | 11531621 | Johansen | Acta Obstet Gynecol Scand | 2001 | cervical intraepithelial neoplasia grade III |
| co-infection (chlaymdia) | 7742335 | Seigel | J Adolesc Health | 1995 | co-infection (chlamydia) |
| keratoconjunctivitis | 18064057 | Kawashima | Eye (Lond) | 2009 | Corneal perforation |
| deferentitis | 2256383 | Kornelishin | Vestn Dermatol Venerol | 1990 | deferentitis |
| arthritic | 16136359 | Phupong | Arch Gynecol Obstet | 2005 | DGI in pregnancy |
| pid/ep | 26656441 | Moore | STD | 2016 | Ectopic pregnancy |
| ectopic pregnancy | 8641479 | Ankum WM | Fertil Steril | 1996 | Ectopic pregnancy |
| ectopic pregnancy | 663716 | Kallenberger | South Med J | 1978 | Ectopic pregnancy |
| rash | 6437201 | Fernandez | Am Heart J | 1984 | endocarditis + death |
| rash | 2000895 | Jackman | Am J Med Sci | 1991 | endocarditis + death |
| terminal | 2000895 | Jackman | Am J Med Sci | 1991 | endocarditis + death |
| endocarditis | 2000895 | Jackman | Am J Med Sci | 1991 | endocarditis + death |
| endocervical mucopus | 9849555 | Chandeying | STI | 1998 | Endocervical mucopus |
| endocervicitis | 2256383 | Kornelishin | Vestn Dermatol Venerol | 1990 | endocervicitis |
| endometritis | 2256383 | Kornelishin | Vestn Dermatol Venerol | 1990 | endometritis |
| Endometritis - salpingitis | 3598229 | Plummer | J Infect Dis | 1987 | Endometritis - salpingitis |
| endomyometritis | 2256383 | Kornelishin | Vestn Dermatol Venerol | 1990 | endomyometritis |
| epididymo-orchitis (EO) | 28705094 | Mensforth | Int J STD AIDS | 2018 | epididymo-orchitis (EO) |
| epidural abscess | 14709128 | van Hal | Med J Aust | 2004 | Epidural abscess |
| epidydimitis | 2256383 | Kornelishin | Vestn Dermatol Venerol | 1990 | epididymitis |
| fear of loss of relationship | 17382038 | Cunningham | Sex Health | 2007 | fear of loss of relationship |
| Female infertility | 28007229 | Tsevat | Am J Obstet Gynecol | 2017 | Female infertility |
| arthritis/tenosynovitis | 141916 | McCord | Arch Intern Med | 1977 | Fever |
| funiculitis | 2256383 | Kornelishin | Vestn Dermatol Venerol | 1990 | funiculitis |
| gingivitis | 10863372 | Holmstrup | Ann Periodontol | 1999 | gingivitis |
| glandular fever | 28703328 | De Marzo | Prostate | 2017 | glandular fever |
| endocarditis | 30021740 | noor | BMJ Case Rep | 2018 | Glomerularnephritis |
| haematuria | 380776 | Amarasuriya | Br J Vener Dis | 1979 | Haematuria |
| hiv | 8652708 | Beck | Int J STD AIDS | 1996 | HIV in Men |
| hiv | 27163174 | Beymer | J Acquir Immune Defic Syndr | 2016 | HIV in MSM |
| hiv | 28608786 | Barbee | STD | 2017 | HIV in MSM |
| hiv | 30976788 | Newman | Clin Infect Dis | 2019 | HIV in women |
| hiv infection | 24713228 | Peterman | Int J STD AIDS | 2015 | HIV in women |
| hiv/aids | 8442924 | Laga | AIDS | 1993 | HIV in women |
| hiv | 8652708 | Beck | Int J STD AIDS | 1996 | HIV in women |
| chlamydia trachomatis | 30228109 | Warr | Sex Transm Infect | 2019 | Infant death |
| Chinese infertility populations | 26688510 | Han | Andrologia | 2016 | Infertility (men) |
| invasive cancer | 8314322 | de Sanjose | Int J Cancer | 1994 | invasive cervical cancer |
| keratoconjunctivitis | 4016048 | Schwab | Br J Ophthalmol | 1985 | keratoconjunctivitis |
| pain | 7813139 | Biro | Clin Pediatr | 1994 | Lack of knowledge about gono |
| ureaplasma urealyticum | 17806051 | Yokoi | CID | 2007 | *M. genitalium / U. urealyticum.* |
| meningitis | 1907402 | Billings | STD | 1991 | meningitis |
| arthritis/tenosynovitis | 141916 | McCord | Arch Intern Med | 1977 | Migratory arthralgias |
| neisseria gonorrhoeae septic arthritis | 28844395 | Rouanes | Rev Med Interne | 2018 | Monoarthritis |
| neonatal conjunctivitis | 2228841 | Lepage | J Antimicrob Chemother | 1990 | neonatal conjunctivitis |
| endocarditis | 6415361 | O'Brien | Medicine (Baltimore) | 1983 | Numerous |
| endocarditis | 23920397 | Belkacem | Sex Transm Infect | 2013 | Numerous |
| bacteremia | 1185739 | Gelfand | J Rheumatol | 1975 | Numerous |
| visual acuity | 3777076 | Wan | Am J Ophthalmol | 1986 | Numerous |
| Stenosis | 1462439 | Kleist | Ugeskr Laeger | 1992 | Numerous |
| obstructive azoospermia (OA) | 26688510 | Han | Andrologia | 2016 | Obstructive azoospermia |
| orchiepidydimitis | 2256383 | Kornelishin | Vestn Dermatol Venerol | 1990 | epididymo-orchitis (EO) |
| haemorrhage | 7607387 | Schneider | Geburtshilfe Frauenheilkd | 1995 | Ovarian capsule hemorrhage |
| epidural abscess | 22578237 | Low | J Neurosurg Spine | 2012 | Paravertebral abscess |
| acute abdomen | 16572933 | Gurlich | Prague Med Rep | 2005 | pelveoperitonitis |
| pericardial effusion | 2317408 | Wilson | Br Heart J | 1990 | Pericardial Effusion |
| tamponade | 2403406 | Coe | Arthritis Rheum | 1990 | Pericarditis |
| pericarditis | 2403406 | Coe | Arthritis Rheum | 1990 | Pericarditis + tamponade |
| fistulae | 1009418 | Osoba AO | Br J Vener Dis | 1976 | Perineal urinary fistulae |
| haemorrhage | 7607387 | Schneider | Geburtshilfe Frauenheilkd | 1995 | peritoneal hemorrhage |
| PID | 29136127 | Reekie | Clin Infect Dis | 2018 | PID |
| Pelvicinflammatorydisease/PID | 8885069 | Kamwendo | Sex Transm Dis | 1996 | PID |
| pid/ep | 26656441 | Moore | STD | 2016 | PID/EP |
| polyarthritis | 6467867 | Rajapakse | Clin Rheumatol | 1984 | Polyarthritis |
| arthritis-dermatitis | 6648748 | Weiner | Sex Transm Dis | 1983 | Popliteal cyst |
| prematurity | 1010761 | Amstey | J Am Vener Dis Assoc | 1976 | Premature rupture of membranes |
| Preterm birth | 24133786 | Waight | J La State Med Soc | 2013 | Preterm birth |
| prematurity | 1010761 | Amstey | J Am Vener Dis Assoc | 1976 | Prolonged rupture of membranes |
| Prostatic carcinoma | 1179565 | Heshmat | Urology | 1975 | Prostate cancer |
| copd | 28013414 | Wang | Eur J Clin Microbiol Infect dis | 2017 | Prostate cancer |
| prostatitis | 2256383 | Kornelishin | Vestn Dermatol Venerol | 1990 | prostatitis |
| prostatovesiculitis | 2256383 | Kornelishin | Vestn Dermatol Venerol | 1990 | prostatovesiculitis |
| arthralgia | 141916 | McCord | Arch Intern Med | 1977 | Reiter's syndrome |
| polyarthritis | 10347969 | Attorri | Medicina (B Aires) | 1998 | Rhabdomyolysis (RML) |
| rhabdomyolysis (RML) | 10347969 | Attorri | Medicina (B Aires) | 1998 | Rhabdomyolysis (RML) |
| genital tract infection | 1530018 | SOper | Am J Obstet Gynaecol | 1992 | Salpingitis |
| genital tract infection | 3598229 | Plummer | JID | 1987 | Salpingitis +/- endometritis |
| septic arthritis | 412257 | Terho | Sex Transm Dis | 1977 | septic arthritis |
| stigmatisation | 17382038 | Cunningham | Sex Health | 2007 | stigmatisation |
| Low birth weight | 28396556 | Moodley | Sex Transm Infect | 2017 | Stillbirth |
| skin manifestation | 7214122 | Owino | Br J Vener Dis | 1981 | subcutaneous abscess |
| subcutaneous gonococcal abscess | 7214122 | Owino | Br J Vener Dis | 1981 | subcutaneous abscess |
| synovial cyst | 412257 | Terho | Sex Transm Dis | 1977 | synovial cyst rupture |
| tamponade | 2403406 | Coe | Arthritis Rheum | 1990 | tamponade |
| genital ulcer diseases | 10858716 | Matteelli | STI | 2000 | Urethral discharge |
| urethritis | 412257 | Terho | Sex Transm Dis | 1977 | Urethritis |
| pelvic infection | 15635136 | Shapiro | Acad Emerg Med | 2005 | urinary frequency, dysuria, urgency |
| genital ulcer diseases | 10858716 | Matteelli | STI | 2000 | Vaginal discharge |
| vasculitic | 17213353 | Jain | J Clin Path | 2007 | Vasculitis |
| invasive vulvar cancer | 1655066 | Sherman | Epidemiology | 1991 | Vulval ca |
| in situ vulvar cancer | 1655066 | Sherman | Epidemiology | 1991 | Vulval ca |

*Most likely site of primary infection was assigned to allow for mapping on figure 5.
